# Identification of Logic Relationships between Genes and Subtypes of Non-Small Cell Lung Cancer

**DOI:** 10.1371/journal.pone.0094644

**Published:** 2014-04-17

**Authors:** Yansen Su, Linqiang Pan

**Affiliations:** Key Laboratory of Image Information Processing and Intelligent Control, School of Automation, Huazhong University of Science and Technology, Wuhan, Hubei, China; Harbin Medical University, China

## Abstract

Non-small cell lung cancer (NSCLC) has two major subtypes: adenocarcinoma (AC) and squamous cell carcinoma (SCC). The diagnosis and treatment of NSCLC are hindered by the limited knowledge about the pathogenesis mechanisms of subtypes of NSCLC. It is necessary to research the molecular mechanisms related with AC and SCC. In this work, we improved the logic analysis algorithm to mine the sufficient and necessary conditions for the presence states (presence or absence) of phenotypes. We applied our method to AC and SCC specimens, and identified 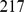 lower and 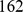 higher logic relationships between genes and two subtypes of NSCLC. The discovered relationships were independent of specimens selected, and their significance was validated by statistic test. Compared with the two earlier methods (the non-negative matrix factorization method and the relevance analysis method), the current method outperformed these methods in the recall rate and classification accuracy on NSCLC and normal specimens. We obtained 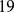 biomarkers. Among 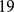 biomarkers, 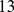 genes have been used to distinguish AC from SCC in practice, and other six genes were newly discovered biomarkers for distinguishing subtypes. Furthermore, *NKX2-1* has been considered as a molecular target for the targeted therapy of AC, and 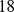 other genes may be novel molecular targets. By gene ontology analysis, we found that two biological processes (‘epidermis development’ and ‘cell adhesion’) were closely related with the tumorigenesis of subtypes of NSCLC. More generally, the current method could be extended to other complex diseases for distinguishing subtypes and detecting the molecular targets for targeted therapy.

## Introduction

Lung cancer is the leading cause of cancer-related deaths in the world [1]. It has been divided into two classes by the World Health Organization (WHO): non-small cell lung cancer (NSCLC) and small cell lung cancer (SCLC) [2]. NSCLC, which has two major subtypes: adenocarcinoma (AC) and squamous cell carcinoma (SCC), accounts for more than a half of all lung cancer cases [2]. However, less than 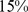 of NSCLC patients survive beyond five years [3]. The limited effectiveness of the diagnosis and treatment of NSCLC is mainly caused by the difficulty to distinguish the subtypes and the limited knowledge about the pathogenesis mechanisms of subtypes of NSCLC.

NSCLC is a system disease, and the difference of AC and SCC may be reflected on the cellular and molecular level. Traditional methods rely on visual cell morphology (e.g. size of tumor and histological features) to distinguish subtypes, which are based on cellular level [4]–[6]. It has been proposed that traditional methods could effectively distinguish SCLC from NSCLC because of the clear distinction between the morphology of SCLC cells and that of NSCLC cells [7]. However, the morphological difference among the subtypes of NSCLC remains unclear [8]. Multiple molecular level data (mRNA, microRNA and methylation data) between NSCLC and normal have been used for analyzing dysfunctions of NSCLC [9]. It was suggested that the discriminating ability of genes obtained by mRNA data was significant greater than those by microRNA and methylation data. Therefore, it is reasonable to retrieve valuable genes and biological processes that have great discriminating ability between AC and SCC on the mRNA level.

A targeted therapeutic agent is designed to interfere with a specific molecular target which plays a crucial role for tumor growth and progression [10]. For example, 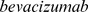, which is a targeted therapeutic agent for the targeted therapy of NSCLC, is a monoclonal antibody for *VEGF*. The gene *VEGF* is crucial because it is higher expressed in lung cancer than in normal lung [11]. Hence, the molecules which play distinct roles between cancer and normal may be important for selecting therapeutic agents. Although targeted therapy shows clinical benefits, targeted agents have not enabled targeted therapies to change clinical outcome dramatically. Moreover, existing targeted therapeutic schedules may be suitable for the prognostic of a special subtype of NSCLC. For example, only patients with non-SCC are better to use 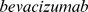 [12]. Therefore, it is necessary to research the molecular mechanisms that are related with the subtypes of NSCLC, to develop effective methods to distinguish AC from SCC and novel therapeutic agents special for the subtypes of NSCLC.

The expression patterns of several genes are found to be special for the subtypes of diseases. For example, the *NKX2-1* gene is expressed in lung AC [13]. The knockdown of *NKX2-1* results growth inhibition in lung AC cell. Therefore, the presence of lung AC depends on the expression of *NKX2-1* [14]. Another example is involved in the research of esophageal cancer, the combination of the genes *GATA6* and *SPRR3* may discriminate among normal epithelium, Barrett's dysplasia and Barrett's esophagus associated AC [15]. Some special relationships exist between the gene pair (*GATA6* and *SPRR3*) and the phenotypes of esophageal cancer. Such examples suggest the existence of relationships between genes and the subtypes of diseases.

The methods that indirectly identify gene-phenotype relationships can be roughly divided into three common steps: construct a gene-gene (or protein-protein) network and a phenotype-phenotype network by pooling interaction data from several databases; connect the gene-gene (or protein-protein) network with the phenotype-phenotype network; use an algorithm (e.g., random walk with restart on heterogeneous network algorithm) to infer pairwise gene-phenotype relationships [16], [17]. However, the noise from the integration of data limits the effectiveness of the detection of gene-phenotype relationships.

Many methods have been developed to directly associate single molecules to phenotypes. The nonnegative matrix factorization (NMF) method is a dimensionality-reducing algorithm to obtain a set of metagenes and associated coefficients [18]. Each phenotype corresponds to a metagene. The coefficient of a gene in a metagene represents the closeness of the relationship between the gene and the phenotype corresponding to the metagene. This method requires to filter several data to ensure the nonnegative condition, which may loss some useful information. Linear correlation coefficients were used to measure genotype-phenotype associations between single proteins in a microbe and the microbe's phenotypes [19]. Slonim et al. used the relevance analysis method (RA) to infer gene-phenotype relationships by estimating mutual information [20]. However, phenotype traits are often influenced not by a single gene, but by combinations of genes. Association rule mining (ARM) is a data mining technique to extract if-then rules with the general form 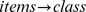 [21]. Bowers et al. designed the logic analysis method to obtain if-then rules from an item or a combination of items to another one. Previous studies have been done to infer logic relationships among genes or proteins using pairwise and triplet logic analysis on expression data or phylogenetic profiles [22]. However, if-then rules may not have many biological cases unless the converse relation holds as well [23].

In this paper, we improve the logic analysis method to mine the necessary and sufficient conditions for the presence states (presence or absence) of phenotypes [22]. The current method takes into consideration both a single gene and a gene pair which may influence phenotypes. We apply the method to infer gene-subtype relationships based on AC and SCC specimens. It is suggested that the expression patterns (expression or no-expression) of identified genes are necessary and sufficient conditions for the presence states of AC or SCC. The effectiveness of the current method is demonstrated on NSCLC and normal specimens. Our results show that the current method outperforms the two existing methods (the NMF method and the RA method) in recall rate and classification accuracy. This work could help to find the biomarkers to distinguish the subtypes of diseases and to design novel targeted therapeutic agents for diseases, as well as reveal the biological processes which are closely related with diseases.

## Results

We applied our method to identify relationships between genes and two major subtypes of NSCLC (AC and SCC). Further, the performance comparison of our method with those of the two earlier methods (the NMF method and the RA method) was made by comparing two measures (the recall rate and classification accuracy) on the data of GSE18842 which contains similar numbers of NSCLC and normal specimens. The biomarkers as well as biological processes which were closely related with the subtypes of NSCLC could be obtained from several interesting relationships between genes and subtypes of NSCLC.

### Identification of gene-subtype lower and higher logic relationships

Given that the number of AC specimens (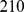) was much larger than that of SCC specimens (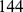) (Table 1), we randomly selected the fixed number (i.e.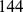) of AC specimens to ensure the similar number of specimens for different phenotypes. We exacted the columns of binary probe data as well as those of phenotype profile data, which correspond to the selected AC specimens and all of the SCC specimens. The new binary probe data and phenotype profile data were formed by the exacted columns of binary probe data and phenotype profile data, maintaining the relative positions of columns. The new binary probe data had size 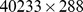, where the first 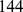 columns corresponded to AC specimens, and the last 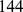 columns refered to SCC specimens. The new phenotype profile data had size 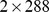, where the first row represented AC and the second one represented SCC. For convenience, we defined the first and second row of the new phenotype profile data as AC profile data and SCC profile data, respectively. The subtypes of NSCLC data comprised the new binary probe data and the new phenotype profile data. We applied our method to the subtypes of NSCLC data to mine gene-subtype logic relationships.

**Table 1:** Data source.

Subtype	No.(n)			
AC	GSE10245(40)	GSE37745(106)	GSE18842(14)	GSE28571 (50)
SCC	GSE10245(18)	GSE37745(66)	GSE18842(32)	GSE28571 (28)
Normal	—	—	GSE18842(45)	—

‘No.’ is the accession number from the Gene Expression Omnibus (GEO) database in NCBI; ‘n’ is the number of specimens; ‘—’ means there are no specimens from the corresponding data set.

#### Identification of probe-subtype lower and higher logic relationships

Based on the subtypes of NSCLC data, we calculated the uncertainty coefficient for a subtype of NSCLC predicted by a probe (or a probe pair), as well as the uncertainty coefficient for a probe (or a probe pair) predicted by the subtype in the reverse direction. The same procedure was applied to random binary probe data and phenotype profile data. The maximum random uncertainty coefficients for logic pairwise and triplet combinations were used as the thresholds for lower and higher logic relationships, respectively. That is, the association of a probe or a probe pair with a subtype was considered significant if and only if its uncertainty coefficients in both directions were found to be greater than the maximal value obtained from the random data. Let 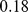 and 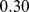 be the thresholds of lower and higher logic relationships, respectively. We obtained 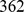 logic pairwise combinations and 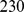 logic triplet combinations with uncertainty coefficients higher than 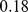 and 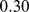, respectively.

Because the significance of the discovered logic pairwise and triplet combinations cannot be exactly verified by the limited knowledge of gene-subtype interactions, a statistical analysis is deserved to be estimated [24]. Suppose the significance level was 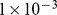. The p-values were all zeros for the discovered logic pairwise and triplet combinations, which were smaller than the significance level. The results of the statistical analysis showed that the discovered logic pairwise and triplet combinations did not interact randomly.

Next, we evaluated the false discovery rate (FDR) to control the global significance of the discovered logic pairwise and triplet combinations. Both FDR values for discovered pairwise and triplet combinations were zero, therefore all of the discovered logic pairwise and triplet combinations were not generated by chance and all of them might represent real associations.

In addition, we calculated the recurrence rate of discovered logic pairwise and triplet combinations among all random trials. The logic relationships with the recurrence rate larger than 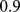 were considered as the relationships which were independent of the specimens selected. Finally, we derived 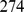 probe-AC lower logic relationships and 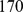 probe-AC higher logic relationships (Table A and B in Table S1).

Note that the AC profile data and SCC profile data were binary complementary vectors. If a probe (or a probe pair) is related with AC by the 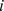
th type of lower (higher) logic relationships, then the probe (the probe pair) is related with SCC by the 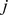
th type of lower (higher) logic relationships, where the uncertainty coefficient of the probe-SCC lower (higher) logic relationship is equal to that of the probe-AC lower (higher) logic relationship, but 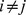. Therefore, the probe which has a close relationship with AC is also closely related with SCC. Finally, we obtained 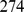 probe-AC/SCC lower logic relationships and 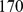 probe-AC/SCC higher logic relationships.

#### Identification of gene-subtype lower and higher logic relationships

Each probe, which was focused on in this paper, is mapped to a single gene. Conversely, a gene may be detected by more than one probe. For example, the *CLCA2* gene was detected by four different probes: *206164_at*, *206165_s_at*, *206166_s_at* and *217528_at*. All of the above four probes were related with AC by the second type of lower logic relationships. Moreover, 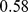, 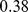, 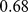 and 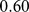 were the mean uncertainty coefficients for each of the four probes related with AC in both directions, respectively. A probe-AC logic relationship set comprised several probe-AC logic relationships, where probes were associated to the same gene. In a probe-AC logic relationship set, the probe-AC/SCC logic relationship with the largest mean uncertainty coefficients in both directions was used to generate a gene-AC/SCC logic relationship as described in Section Materials and Methods. Thus, *CLCA2* was related with AC by the second type of lower logic relationships and the coefficient of the *CLCA2*-AC/SCC relationship was 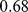.

According to the above method, 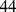 gene-AC/SCC lower logic relationships were generated from 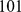 probe-AC/SCC lower logic relationships (Table A in Table S2). Each of the rest 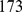 probe-AC/SCC lower logic relationships generated a gene-AC/SCC lower logic relationship. Finally, we obtained 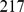 gene-AC/SCC lower logic relationships (Table A in Table S3).

We found that if a gene was detected by more than one probe, and the probes were related with subtypes by lower logic relationships, then the types of the probe-AC/SCC lower logic relationships were the same. It is suggested that the probes which are associated to the same gene may be related with subtypes by the same way.

We obtained six gene-AC/SCC higher logic relationships from 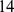 probe-AC/SCC higher logic relationships (Table B in Table S2). Each of the rest 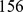 probe-AC/SCC higher logic relationships generated a gene-AC/SCC higher logic relationship. Finally, we obtained 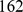 gene-AC/SCC higher logic relationships (Table B in Table S3).

In what follows, we discussed examples of logic relationships which may be inferred from phenomenons previously described in the literature.

#### Examples of gene-subtype lower logic relationships

If each of the genes *DSG3*, *CLCA2*, *DSC3* and *PKP1* was expressed, then SCC was present, while AC was absent. In addition, if each of above genes was not expressed, then SCC was absent and AC was present. That is, the expression of each of above genes was a sufficient and necessary condition of the presence of SCC as well as the absence of AC. Our results suggested that genes (*DSG3*, *CLCA2*, *DSC3* and *PKP1*) may distinguish subtype AC from SCC. Given that intracellular bridges are one of the most characteristic of SCC but not of AC, proteins involved in these bridges may be up-regulated in SCC only, such as desmosome proteins and intercellular junctional proteins [25]. *Desmoglein 3* is the protein encoded by *DSG3*. This protein is a calcium-binding transmembrane glycoprotein component of desmosome in vertebrate epithelial cells. The protein encoded by *DSC3* is a calcium-dependent glycoprotein (*Desmocollin 3*) that is required for cell adhesion and desmosome formation. The protein encoded by *PKP1* may be involved in molecular recruitment and stabilization during desmosome formation. The protein encoded by *CLCA2* belongs to the calcium sensitive chloride conductance protein family. It may serve as adhesion molecule for lung metastatic cancer cells. The above four genes (*DSC3*, *DSG3*, *PKP1* and *CLCA2*) which are associated to desmosomes were found to be up-regulated in SCC compared to the AC subtype [26]. Concretely, *DSG3* showed high expression in SCC, while low expression in AC [26]. *DSC3* was also upregulated in SCC exclusively [27], [28]. In primary lung tumors, *DSC3* was a potential diagnostic marker for lung squamous cell carcinoma [29]. *PKP1* showed a 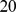 times greater level of expression in SCCs than in ACs and normal lung and thus may be useful in histopathological diagnosis [28]. *CLCA2* has been inferred to be specifically overexpressed in SCC [30].

We found that subtype AC (SCC) was present (absent) if and only if *NKX2-1* was expressed. It is inferred that the expression of *NKX2-1* in the specimen of AC is much higher than that of SCC. *NKX2-1* which is known as thyroid transcription factor 1 (*TITF-1*) is a homeodomain-containing transactivating factor, and it expressed in the terminal lung bronchioles and lung periphery predominantly [31]. The presence of *NKX2-1* protein was prevalent in AC, while in SCC *NKX2-1* was absent [13]. It is in accordance with our results.

#### Examples of gene-subtype higher logic relationships

The higher logic relationships between gene pairs and SCC were selected for further analysis. Gene pairs (*GPX2*, *ITGB8*) and (*GPX2*, *SLC2A12*) were related with SCC, via an ‘AND’ logical relationship (higher logic relationship type 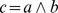). It indicates that *GPX2*, *ITGB8* and *SLC2A12* were all expressed if the specimen was SCC. Moreover, all of the genes *GPX2*, *ITGB8* and *SLC2A12* were not expressed if the specimen was AC. *GPX2* was detected to have higher expression in SCC compared with AC and normal [32], [33]. We were unaware of evidence in the literature of the relationships between *ITGB8*, *SLC2A12* and the subtypes of NSCLC. Our analysis generated several novel relationships.

There are not enough evidences for higher logic relationships to distinguish the subtypes of NSCLC. Hence, most of the relationships between gene pairs and the subtypes of NSCLC have not been confirmed. As the lack of knowledge about the regulation relationships between genes and subtypes, the exact relationships between the common gene pairs and subtypes are deserved to be checked.

### Performance comparison

We exacted the columns of binary probe data as well as those of phenotype profile data, which correspond to the 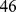 NSCLC specimens and 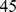 normal specimens of GSE18842. The new binary probe data and phenotype profile data were formed by the exacted columns of binary probe data and phenotype profile data, maintaining the relative positions of columns. The NSCLC and normal data comprised the new binary probe data and phenotype profile data.

#### Application of the three methods

We firstly applied the current method to the NSCLC and normal data. We set the 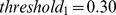, and obtained 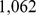 probe-phenotype lower logic relationships. The significance and global significance of the discovered relationships were verified by statistic test.

Next, we applied the NMF method to the NSCLC and normal data. Rows with 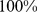 ‘
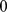
s’ were filtered from the binary probe data to ensure the feasibility of the NMF method. The rest binary probe data contained 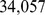 rows and 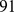 columns. Because two clusters of specimens (AC and SCC) were included in the binary probe data, we chose 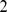 as the dimensionality reduction parameter 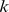 for the NMF method. Among the obtained two metagenes, the second metagene had higher expression level in almost all (i.e. 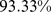) of the NSCLC specimens, while lower expression level in almost all (i.e. 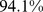) of the normal specimens. The probes within the second metagene were sorted according to their activation levels (Table S4). The first probe represented the most closely related probe to the NSCLC phenotype, while the last probe represented the least closely related probe.

Finally, we applied the RA method to the NSCLC and normal data. We sorted the probes by the mutual information between the probe profiles and NSCLC profiles.

Note that the correlations between gene pairs and phenotypes could be measured by the current method, but they could not be measured by the NMF and RA methods. Hence, from this point of view, the current method is superior to the two earlier methods. All of the three methods could find single genes closely related with phenotypes. Hence, we just identified the gene-phenotype lower logic relationships by the current method and compared the results with those obtained by the two earlier methods.

#### Performance comparison for the three methods

We selected two datasets involved the genes which are related with NSCLC. One dataset contains 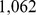 high frequency genes on the mRNA level detected by Huang et al. (Table S5) [9]. It was showed that these genes belonged to the top 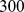 dysfunctional gene sets with good discriminating ability. We chose the dataset because it was collected from GEO with the accession number GSE18842, which was also the source of the NSCLC and normal data in this work. The other dataset contains 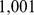 up-/down-regulated genes found by Urgard et al., where 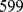 genes are down-regulated and 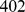 genes are up-regulated in NSCLC compared to the normal tissue (Table S5) [34]. A total of 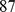 genes were shared by the above two datasets. Because it is hard to validate the genes included in each dataset, it is reasonable to consider these 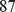 genes as the truth data to estimate the performance of different methods in this work.

In order to estimate the performance of the current method and compare its performance with the two earlier methods (the NMF method and the RA method), we calculated a measure: the recall rate which was the ratio of the number of detected genes in the truth data to the total number of genes in the truth data. Note that the recall rate may be biased by the incomplete nature of the truth data. Further, we evaluated the classification accuracy which evaluated the discriminating ability of resulted probes.

Among all of the genes detected by 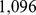 probes obtained by the current method, 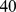 genes were in the truth data. Hence, the recall rate of the current method was 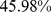. To compare the recall rate of the current method with those of the two earlier methods, we selected the top 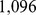 probes obtained by the NMF method and the RA method, respectively. We found 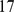 and zero of the genes in the truth data have been detected by the NMF method and the RA method, respectively. Hence, the recall rate of NMF and RA were 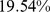 and 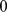, respectively. The current method had higher recall rate than NMF and RA.

**Figure 1:**
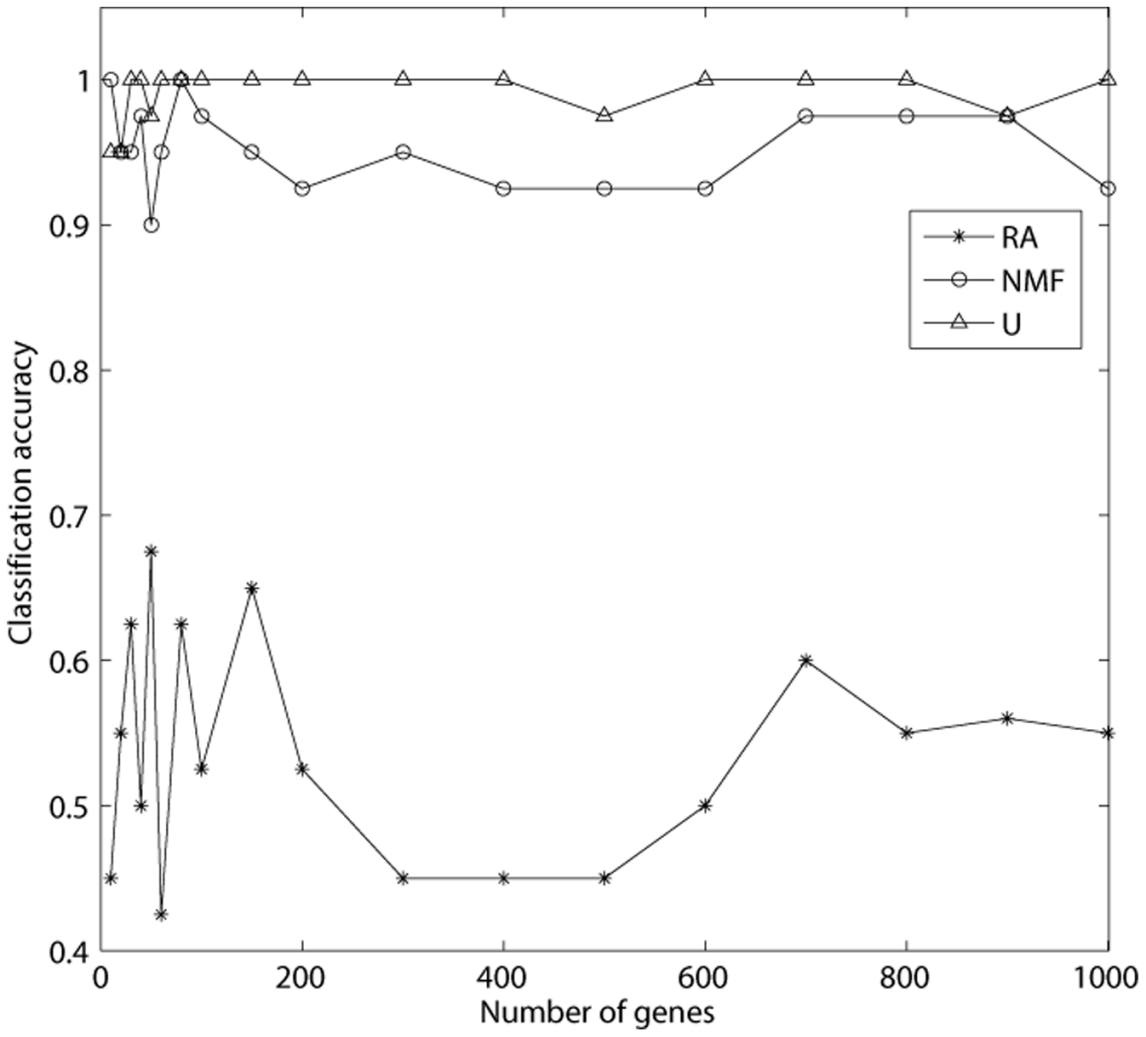
The recall rate of genes obtained by three methods. According to each method, we rank the genes in descending order by the coefficients of genes related with phenotypes. We selecte the top 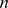 genes, where 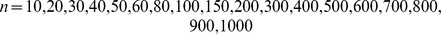. The classification accuracy is calculated based on the top 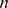 genes. ‘RA’, ‘NMF’ and ‘U’ represent the relevance analysis method, the non-negative matrix factorization method and the current method, respectively.

By Fig. 1, we found that the current method achieved higher classification accuracy than the NMF method and the RA method. Additionally, the average classification accuracy of our method approached to 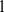 (i.e. 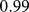), which means that the probes obtained by our method has a great classification ability. In the figure, each curve was steady with little fluctuation. It indicates that the classification accuracy was little sensitive to the number of probes.

### Biomarkers and key gene pairs

#### Biomarkers inferred by gene-subtype lower logic relationships

In previous research, a total number of 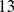 genes have been reported to be used to differentiate between AC and SCC, and these genes are *DSG3* [26], *CLCA2* [30], *DSC3* [27], *PKP1* [28], *NKX2-1* [35], GJB5 [26], KRT6B [36], SERPINB13 [36], TP63 [37], TRIM29 [38], *KRT5* [28], *NTRK2* [28] and *DST* [39]. We sorted the genes which were involved in the gene-AC/SCC lower logic relationships in descending order by their coefficients. Interestingly, all of above 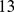 genes were included in the top 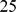 genes. It is suggested that a gene which has high uncertainty coefficient may clearly distinguish AC from SCC.

**Figure 2:**
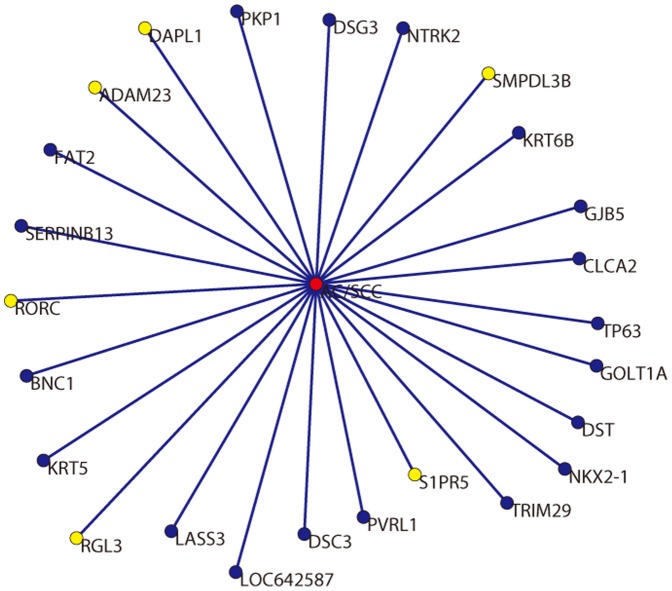
25 genes are related with the subtypes of NSCLC. There are 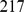 genes related with subtypes of NSCLC by lower logic relationships, and each gene attaches a coefficient. The genes are ranked according to coefficients in descending order. The top 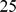 genes are selected to identify biomarkers. The blue nodes represent 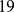 biomarkers identified in this work. The yellow nodes represent six genes which are not related with NSCLC on the NSCLC and normal specimens. The red nodes represent subtypes, i.e. AC and SCC.

To obtain a set of biomarkers, we firstly selected the top 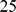 ranked genes (Fig. 2). Because the molecular targets for targeted therapeutic agents play crucial roles for tumor, the biomarkers for targeted therapy should have the distinct biological functions between NSCLC and normal. Next, an intersection set was generated between top 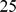 genes and the genes involved in gene-NSCLC lower logic relationships (the genes have been obtained in subsection ‘Performance comparison’). Finally, 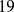 intersect genes were regarded as the biomarkers for distinguishing AC from SCC, as well as novel molecular targets for targeted therapeutic agents. That is, the set of biomarkers comprised *DST*, *CLCA2*, *KRT5*, *DSG3*, *GJB5*, *SERPINB13*, *BNC1*, *TRIM29*, *LOC642587*, *PKP1*, *KRT6B*, *FAT2*, *GOLT1A*, *DSC3*, *NKX2-1*, *TP63*, *LASS3*, *PVRL1* and *NTRK2*.

#### Key gene pairs inferred by gene-subtype higher logic relationships

We grouped together the gene-subtype higher logic relationships with the same logic function. Because the two logic functions AND (Type 1) and XOR (Type 8) have more intuitive biological interpretations than other logic functions, we restricted our analysis to these two logic functions. The key gene pairs were defined as the gene pairs involved in the gene-subtype higher logic relationships with logic function AND or XOR. We obtained 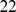 key gene pairs in total, where 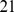 and 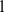 gene pairs were related with AC/SCC through the logic functions AND and XOR, respectively (Table S6). This result may be explained by the strict parameters we chose.

### Gene Ontology analysis

The Gene Ontology (GO) is a structured and controlled vocabularies and classifications about the annotations of genes, gene products and sequences [40]. GO includes three categories of terms: biological processes, molecular functions and cell components. We were focused on the biological processes enriching the genes involved in lower logic relationships. So, in what follows, when we say GO terms, it means the GO terms in the ‘biological process’ category.

According to 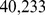 probe-AC/SCC pairwise associations and their uncertainty coefficients, we obtained a gene set containing 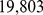 genes without overlap and each gene attached a coefficient. A total of 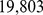 genes were ranked in descending order by coefficients and given as input to the Gorilla. The Gorilla gave 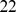 significant GO terms like ‘tissue development’ (GO: 0009888), ‘epidermis development’ (GO: 0008544) , and ‘epithelial cell differentiation’ (GO: 0030855) (Part A in Appendix S1). Given that the significant GO terms were retrieved based on the subtypes of NSCLC data, it has to be checked whether the significant GO terms are also significant on NSCLC and normal specimens. The same procedure was applied to the ranked genes based on the NSCLC and normal data. The test revealed 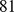 significant GO terms with significant value 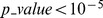 (Part B in Appendix S1). In total, seven out of 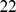 GO terms on the subtypes of NSCLC data were also significant on the NSCLC and normal specimens (Table 2). It indicates that the following seven biological processes are important for tumorigenesis of NSCLC: tissue development, epidermis development, epithelial cell differentiation, anatomical structure development, developmental process, cell adhesion and biological adhesion.

**Table 2:** Significant GO terms.

GO terms	Description	P-value1	P-value2	E1	E2
GO:0009888	tissue development	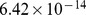	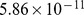	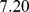	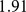
GO:0008544	epidermis development	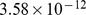	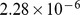	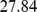	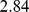
GO:0030855	epithelial cell differentiation	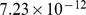	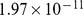	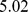	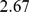
GO:0048856	anatomical structure development	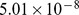	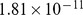	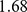	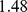
GO:0032502	developmental process	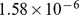	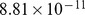	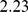	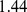
GO:0007155	cell adhesion	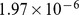	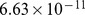	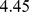	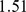
GO:0022610	biological adhesion	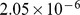	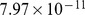	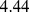	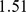

‘P-value1’ and ‘P-value2’ denote the p-value scores of GO terms based on the subtypes of NSCLC data and NSCLC and normal data, respectively. ‘E1’ and ‘E2’ are the enrichment values of GO terms based on the subtypes of NSCLC data and NSCLC and normal data, respectively.

Further, we grouped the genes closely related with the subtypes of NSCLC into two groups by the types of gene-SCC lower logic relationships. We mapped the 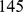 genes which were related with SCC (AC) by Type 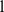 (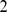) lower logic relationships to GO terms. Gene ontology analysis revealed 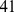 GO terms with the p-value scores smaller than 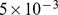 and the enrichment scores larger than 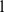. Among 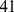 significant GO terms, epithelial cell differentiation (GO: 0030855) and cell adhesion (GO: 0007155) were also involved in the seven significant GO terms which may be important for tumorigenesis of NSCLC. It indicates that dysfunction of epithelial cell differentiation and cell adhesion is important for both of the tumorigenesis of AC and SCC.

In addition, we mapped the 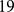 identified biomarkers to GO terms. The resulted significant GO terms were cell adhesion (GO: 0007155) and epidermis development (GO: 0008544) with the p-value scores smaller than 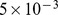 and the enrichment scores larger than 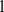. It indicates that genes annotated to epidermis development and cell adhesion may be differently regulated between AC and SCC.

By mapping the 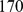 higher logic relationships to GO terms, we obtained 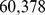 pairs of GO terms, with 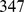 different GO terms. Among all pairs of GO terms, 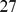 pairs of GO terms involving 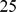 GO terms were significant with the p-value scores smaller than 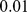, enrichment score larger than one and the number of gene pairs larger than two. These combination of biological processes may be pivotal for differentiating AC and SCC, including a combination of ‘transport’ (GO: 0006979) and ‘regulation of transcription, DNA-dependent’ (GO: 0006355), a combination of ‘oxidation-reduction process’ (GO: 0055114) and ‘nervous system development’ (GO: 0007399), and a combination of ‘negative regulation of cell proliferation’ (GO: 0008285) and ‘muscle contraction’ (GO: 0006936).

## Discussion

In this paper, we improved the logic analysis method to infer sufficient and necessary conditions for the presence states (presence or absence) of a phenotype. The current method omits the integration of networks, and identifies not only gene-phenotype pairwise combinations (i.e. lower logic relationships), but also triplets combinations (i.e. higher logic relationships). On one hand, it avoids the incompleteness of data sources and the noise from the integration of data; on the other hand, the triplets combinations reflect the combination effect of gene pairs on phenotypes, other than an individual effect. Some examples of lower and higher logic relationships demonstrated the biological relevance of our results. However, the accuracy of all discovered logic relationships cannot be verified because of the current limited knowledge of the relationships between genes and phenotypes. The statistics analysis strengthened the reliability of discovered logic relationships. In addition, the current method was compared with the two earlier methods (the NMF method and the RA method). The current method was superior to the two earlier methods because of its ability of mining gene pairs which are closely related with phenotypes. Moreover, the current method gained the higher recall rate and classification accuracy than the two earlier methods. Our results display the advantage of the current method in mining genes closely related with phenotypes.

The discovered gene-subtypes logic relationships in this paper are equivalent relationships between the expression patterns (expression or no-expression) of genes and the presence states (presence or absence) of phenotypes. That is, both a expression pattern of a gene and a presence state of a phenotype must be either simultaneously true or simultaneously false. For example, *DSC3* is expressed if and only if the specimen is SCC, as *DSC3* is related with SCC by the first type of lower logic relationship. If a gene is related with a phenotype by a logic relationship, then either the expression pattern of a gene or the presence state of a phenotype may be determined by the underlying logic relationship. Concretely, given a phenotype, the expression pattern of genes in a phenotype could be determined by the logic relationship. For example, the expression pattern of *DSC3* in SCC depends on the type of *DSC3*-SCC lower logic relationship. Conversely, given a expression pattern of a gene, the presence state of a phenotype could also be determined by the underlying logic relationships.

The type of a discovered gene-AC lower logic relationship was totally different from that of the gene-SCC lower logic relationship, where the genes involved in two relationships are the same. It indicates that the totally different types of lower logic relationships between genes and phenotypes may be the intrinsic reason for the different expression patterns of genes in distinct phenotypes.

A total of 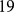 genes identified in our work were regarded as the biomarkers for distinguishing AC from SCC, as well as novel molecular targets for targeted therapeutic agents. Besides the 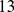 genes identified in the literature (*DST*, *CLCA2*, *KRT5*, *DSG3*, *GJB5*, *SERPINB13*, *TRIM29*, *PKP1*, *KRT6B*, *DSC3*, *NKX2-1*, *TP63*, and *NTRK2*), most of the rest genes (*BNC1*, *FAT2*, *LASS3* and *PVRL1*) are likely to be the novel biomarkers to distinguish AC from SCC. The *BNC1* gene is thought to play a regulatory role in ‘keratinocyte proliferation’, and the *LASS3* gene is participated in ‘keratinocyte differentiation’. Both of the biological process ‘keratinocyte proliferation’ and ‘keratinocyte differentiation’ are children of ‘keratinization process’. Because the genes involved in ‘keratinization process’ are higher expressed in SCC as compared with AC [26], *BNC1* and *PVRL1* which are either a upstream regulatory factor or a member of these high expressed genes may be able to differentiate AC and SCC. *FAT2* functions as a cell adhesion molecular, and it controls cell proliferation. As ‘cell adhesion’ is one of the significantly important biological processes for tumorigenesis of NSCLC, the cell adhesion molecular (*FAT2*) is deserved to be a biomarker to distinguish AC from SCC. Until recently, the function of *LOC642587* and *GOLT1A* has been unknown. Further experimental validation is needed to confirm the differentiating ability of these two genes. In addition, the *NKX2-1* gene has been considered as a novel oncogene [35], and it opens new windows for novel targeted therapies [41]. Although there has limited evidence to confirm the rest 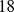 genes to be molecular targets for targeted therapy, these 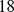 genes provide useful clues for targeted therapy.

By gene ontology analysis, the biomarkers inferred in gene-subtype lower logic relationships were significantly enriched in biological processes of ‘cell adhesion’ (GO: 0007155) and ‘epidermis development’ (GO: 0008544). The identified biological processes had nonrandom probability values and enrichment scores, and they were also significant biological processes which were important for tumorigenesis of NSCLC. The discovered biomarkers in the biological processes ‘cell adhesion’ and ‘epidermis development’ (i.e. *DST*, *CLCA2*, *DSG3*, *PKP1*, *FAT2*, *DSC3*, *PVRL1*, *KRT5*, *GJB5*, *BNC1*) account for more than a half of all discovered biomarkers. The expression of these genes were all sufficient and necessary conditions of the presence of SCC as well as the absence of AC. It indicates that genes annotated to epidermis development and cell adhesion may be differently regulated between AC and SCC. In previous research, several genes involved in ‘cell adhesion’ as well as ‘epidermis development’ were significantly up-regulated in SCC compared to normal and AC [26], which is in accordance with our results. The majority of cell adhesion genes (predominantly desmosomal genes) and epidermis development genes have been found to be significantly up-regulated in SCC compared to normal tissue and the AC subtype. For example, desmosomal genes (DSC3 and DSG3) and epidermis development genes (KRT5) were increased in SCC compared to the AC subtype. Our results strengthen the importance of ‘cell adhesion’ and ‘epidermis development’ in distinguishing AC from SCC. It indicates that cell adhesion genes and epidermis development genes play central roles in the drug delivery and are promising targets for novel therapies.

In conclusion, biomarkers identified in this paper could be used to classify patients for the treatment of NSCLC. A classification based on the discovered biomarkers could help to supply potential information in clinical decision making. The identified gene-subtype logic relationships and GO terms may extend perception to disease mechanisms for NSCLC. In addition, the targeted therapy agents may also be designed to interfere with the discovered biomarkers. However, several biomarkers and GO terms have been less well understood yet, which needs further experimental research.

## Materials and Methods

### Data source and data processing

We use the specimens of GSE10245 (a Gene Expression Omnibus accession number for microarray data), GSE37745, GSE18842 and GSE28571 to form a microarray expression data, which are available from National Center for Biotechnology Information (NCBI, http://www.ncbi.nlm.nih.gov/). Each specimen is annotated with a phenotype property (AC, SCC and Normal) (Table 1). The microarray expression data (see Appendix S2) contains the expression data of 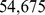 probes in 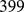 specimens.

The microarray expression data is converted into a binary probe data using the Microarray Suite 5 (Mas5) algorithm [42]. The Mas5 algorithm generates a p-value which assesses the reliability of the expression level for each probe and a detection call which is a three-valued discrete data of a p-value. Specifically, if a p-value is less than 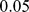, then the detection call is ‘Present’; if a p-value is greater than 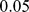 and less than 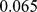, then the detection call is ‘Marginal’; if a p-value is greater than 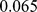, then the detection call is ‘Absent’. Probes are flagged ‘Marginal’ or ‘Absent’ when the detection of probes is not considered to be significantly reliable. Hence, it is reasonable to consider that the probes with flag ‘Marginal’ or ‘Absent’ are not significantly detected. In this work, we turn ‘Marginal’ and ‘Absent’ flags to ‘
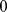
’s, and turn ‘Present’ flags to ‘
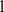
’s. A ‘0’ in the 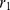
th row and 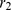
th column of the binary probe data mean the 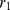
th probe is not detected in the 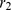
th specimen, while a ‘1’ indicates the probe is detected.

Once converted, the binary probe data is supplemented with an additional phenotype profile data. The phenotype profile data has three rows and 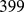 columns. The 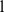
st, 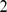
nd and 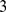
rd rows correspond to AC, SCC and Normal specimens, respectively (Appendix S2). The phenotype profile data represents the properties of phenotypes, where a ‘1’ in the 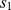
th row and 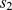
th column of the phenotype profile data means the 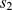
th specimen belongs to the 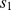
th phenotype, while a ‘0’ means not.

The 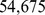 probes are associated to genes according to the information of GPL570 (a microarray chip)(see Table S7). According to the number of genes that a probe detects, probes can be classified into three categories: probes detecting a single gene, probes detecting more than one gene, and probes detecting no genes. In Table S7, there are 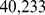 probes associated to a single gene, 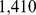 probes associated to more than one gene and 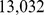 probes associated to no genes. We are focused on the 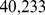 probes associated to a single gene. The binary probe data contains 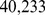 rows, describing the detection patterns of probes.

### Current relationship-inference method

#### Calculating uncertainty coefficient

The vector 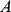 describes the vector 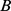 via either Type 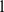 or Type 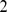 lower logic function (see Table 3), i.e. 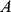 and 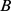 constitute a logic pair. A logic combination of the vectors 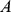 and 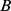 describes the vector 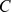 via one of the eight higher logic functions (see Table 4), i.e. 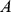, 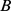 and 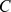 compose a logic triplet. Uncertainty coefficient for a vector pair or a vector triplet is a measure to describe to what extent a vector or a combination of two vectors predicts another vector [22].

**Table 3:** Lower logic function of vector 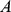.

Type	Symbol	Lower logic function	Logic statement
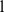	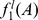	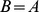	The value of 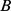 is 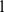 iff the value of 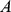 is 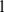
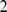	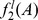	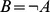	The value of 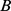 is 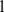 iff the value of 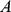 is 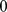

‘
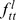
’ denotes the function symbol of type 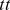 of lower logic relationships, where 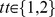 and 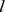 represents the sign for the lower logic relationships.

**Table 4:** Higher logic function of vectors 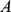 and 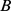.

Type	Symbol	Higher logic function	Logic statement
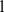	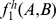	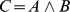	The value of 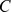 is 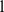 iff the values of both 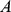 and 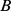 are 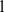
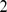	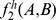	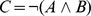	The value of 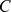 is 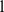 iff the value of 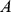 is 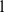 or that of 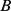 is 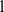
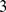	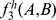	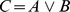	The value of 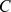 is 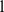 iff the value of 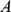 or that of 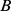 is 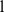
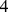	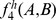	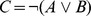	The value of 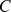 is 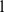 iff the values of both 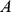 and 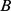 are 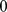
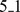	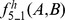	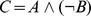	The value of 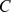 is 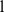 iff the value of 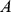 is 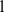 and that of 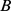 is 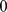
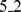	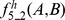	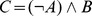	The value of 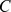 is 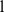 iff the value of 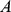 is 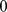 and that of 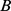 is 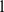
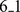	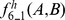	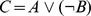	The value of 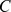 is 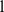 iff the value of 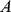 is 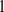 or that of 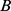 is 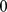
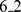	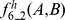	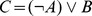	The value of 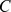 is 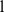 iff the value of 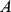 is 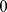 or that of 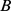 is 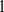
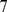	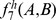	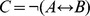	The value of 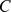 is 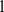 iff either the value of 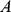 or that of 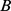 is 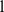
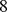	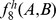	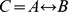	The value of 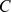 is 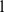 iff the values of both 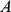 and 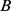 are 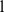 or 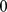

‘
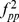
’ denotes function symbol of type 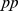 of higher logic relationships, where 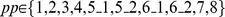 and 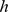 represents the sign for the higher logic relationships.

The value of 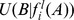 represents how well for the vector 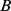 is described by the vector 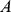 under a lower logic function 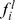, where 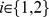, and 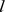 is the symbol for lower logic functions. The value of 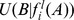 is calculated as follows (Matlab codes available in Appendix S3):
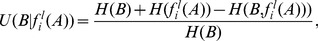
(1)where 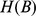 is the entropy of 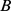, and 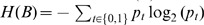, where 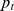 is the probability of occurrence of 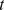, and 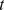 is either 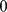 or 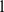. 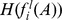 is the entropy of the vector 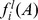. 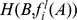 is the joint entropy of 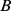 and 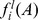, and 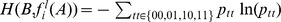, where 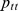 is the probability of occurrence of 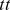.

The uncertainty coefficient for 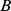 given 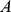, which is denoted by 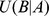, is the maximum of 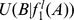 and 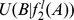. Referring from 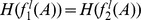 and 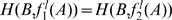, we got 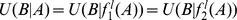. The value of 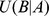 ranges from 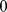 to 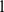, where 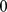 means that 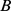 is independent of 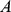, and 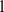 means that 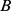 is completely determined by 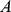.

We calculate the degree to which the logic combination of the vectors 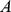 and 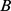 (e.g. 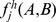) describes a third vector 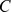 as follows (Matlab codes available in Appendix S3):
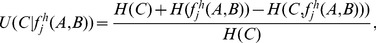
(2)where 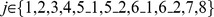; 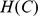 and 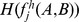 are the entropy of 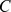 and 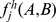, respectively; 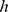 is the symbol for higher logic functions; 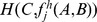 is the joint entropy of 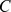 and 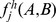.

As similar with 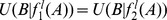, we have

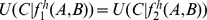
,

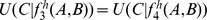
,

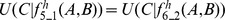
,

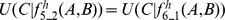
,

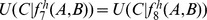
.

The uncertainty coefficient for 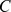 predicted by a logic combination of 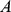 and 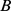 is denoted by 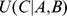. 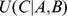 is equal to the maximum of the following five values: 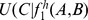, 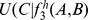, 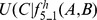, 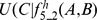, 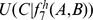. The value of 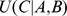 ranges from 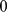 to 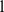.

A well known measure, the confidence, is used to select the greatest possible rules by which probes related with phenotypes from the set of all possible rules [43]. Here, the set of all possible rules are lower/higher logic functions corresponding to the maximum lower/higher uncertainty coefficients. Suppose the vectors 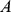 and 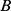 follow the lower logic function 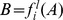, where 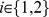. The confidence of 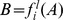 is calculated as: 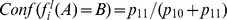, where 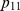 and 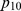 refer to the joint probability of occurrence of 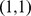 and 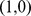 for the vectors 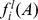 and 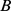, respectively. Suppose vectors 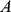, 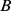 and 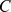 follow the rule 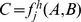, where 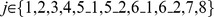, then the confidence of the rule (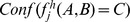) is also the ratio of 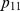 to 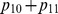, and 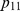 and 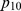 refer to the joint probability of occurrence of 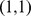 and 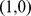 for the vector 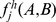 and vector 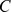. We calculate the confidence for two lower (or higher) logic functions with the same value of 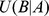 (or 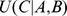). The higher the confidence of a logic function, the higher the probability that vectors follow the logic type corresponding to the logic function.

The value of 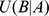 measures how well 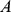 approximates a sufficient condition for 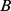, and the value of 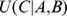 measures how well the combination of 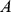 and 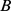 approximates a sufficient condition for 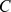. We improve the logic analysis by taking the reverse uncertainty coefficients into consideration. That is, given the 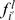 and 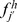 to be the final lower and higher logic functions, respectively, we calculate the value of 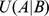 and 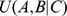 as follows (Matlab codes available in Appendix S3):
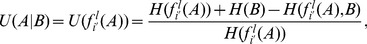
(3)where 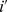 is either 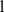 or 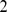, and 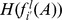, 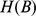 and 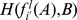 are the same as those in e.q (1).
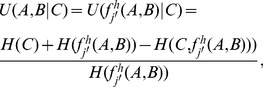
(4)where 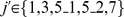; 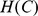 and 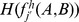 are the entropy of vector 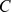 and 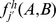, respectively; 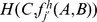 is the joint entropy of vector 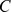 and 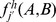.

#### Calculating random uncertainty coefficient

Let 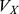 be the histogram of the vector 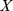. Suppose 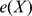 is the set of distinct elements of 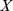. For each 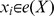, 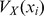 is the number of times 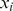 appears in 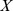, where 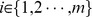, and 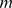 is the number of elements in 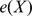 [44].

Given the vectors 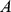 and 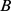, the random uncertainty coefficient 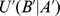 and 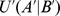 is calculated using the following steps:

1. Generate random vectors 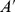 and 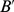. 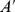 and 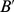 maintain the same distribution of the vectors 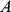 and 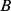 (i.e., 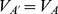, 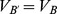).
2. Compute 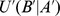, where 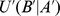 is the uncertainty coefficient for 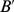 given 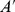 in a trial.
3. Compute 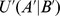, where 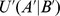 is the uncertainty coefficient for 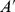 given 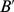 in a trial.

The calculation of 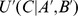 and 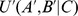 involves the following three steps:

1. Generate random vectors 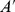 and 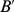, maintaining the individual distribution and pairwise distribution. The vector 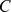 retains the position of its elements unchangeably. Note that 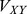 could determine 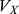 and 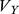. We generate 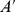 and 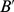 maintaining 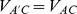 and 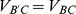.
2. Compute 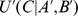, where 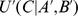 is the uncertainty coefficient for 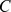 given the combination of 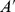 and 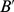 in a trial.
3. Compute 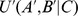, where 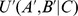 is the uncertainty coefficient for the combination of 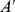 and 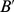 given 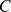 in a trial.

#### Identification of probe-phenotype lower and higher logic relationships

Thresholds are defined here to separate lower or higher logic relationships from logic pairwise or triplet combinations. Let 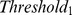 and 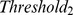 be the thresholds of lower and higher logic relationships, respectively. We calculate the random uncertainty coefficients of all probe-phenotype pairwise (i.e. a probe and a phenotype) and triplet combinations (i.e. a probe pair and a phenotype). 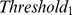 is the maximum uncertainty coefficient of all probe-phenotype pairwise combinations, and 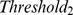 is the maximum uncertainty coefficient of all probe-phenotype triplet combinations.

If the values of both 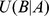 and 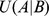 are higher than 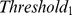, then 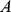 approximates a necessary and sufficient condition for 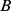. There exists a lower logic relationship between 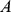 and 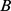. If the values of both 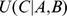 and 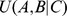 are higher than 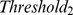, and there are no lower logic relationships between either 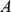 or 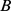 and 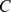, then the logic combination of vector 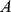 and 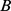 approximates a necessary and sufficient condition for 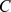. There is a higher logic relationship between the combination of 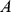 and 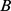 and 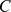.

#### Statistical analysis

A p-value is defined as a measure to reflect how well vectors related in the form of discovered logic pairwise or triplet combinations compared to chance relations. Here, an actual uncertainty coefficient is compared to the random one in a random trial. The p-value of a discovered logic pairwise or triplet combination is equal to the number of random trials, in which either one of the two random uncertainty coefficients of pairwise or triplet combination of random vectors in both directions is higher than the actual one, divided by the total number of random trails.

Specifically, we compare 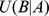 and 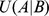 to the distribution of 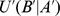 and 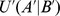, where 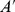 and 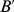 are the random vectors of 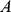 and 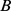. For each pair of 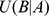 and 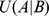, we calculate the random uncertainty coefficients 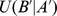 and 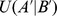 in 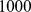 random trails. We have the p-value of the discovered logic pairwise combination: 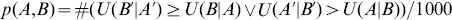, where 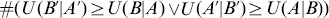 means the number of random trials in which either one of the following two items 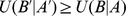 and 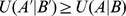 is tenable. Similarly, the p-value of the discovered logic triplet combination is 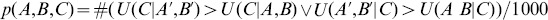, where 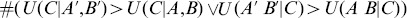 means the number of random trials in which either 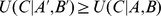 or 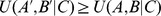 is tenable.

#### False discovery rate

In order to evaluate a global significance value of the actual discovered logic pairwise or triplet combinations, we measure a false discovery rate (FDR) [45]. Given the threshold of lower logic relationships, we estimate the number of discovered random logic pairwise combinations with the mean uncertainty coefficients larger than the threshold by chance. We generate 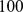 random independent data and extract discovered random logic pairwise combinations from each random data. The estimated number of false discovered logic pairwise combinations (denoted as 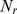) is calculated as the mean number of discovered random logic pairwise combinations obtained from these 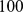 random independent data. If 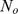 is the number of actual discovered logic pairwise combinations, then 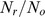 is a simple estimated positive FDR for the given threshold. We can scan all probe-phenotype pairwise combinations, but it take too much time to scan all triplet combinations. Therefore, we randomly select a fixed number of triplet combinations (e.g. 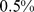 of all possible triplet combinations) and extract higher logic relationships with respect to actual and random (denoted as 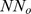 and 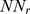), respectively. This process is repeated for 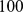 times, and the FDR is the mean value of 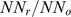. The Matlab codes are available in Appendix S3.

#### Cross validation

In a random trial, a fixed number of columns corresponding to each phenotype are selected from the original probe binary data and phenotype data to form the random probe binary data and random phenotype data. We check whether a logic relationship could be obtained in the random trial. The above processes are repeated for 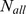 times, where 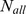 represents the number of all random trials.

The recurrence rate Q is used to evaluate the reliability of logic relationships as follows:
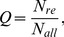
(5)where 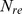 represents the number of recurrance times of a logic relationship in all random trials, and 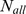 is the number of all random trials.

### Mapping probe-phenotype relationships to gene-phenotype relationships

On the basis of lower and higher probe-phenotype logic relationships, lower and higher gene-phenotype logic relationships are generated as follows.

Suppose all the probes detecting genes 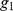, 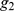 and 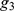 form a set 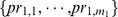, 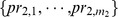 and 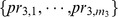, where 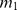, 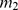 and 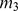 are the size of the set and 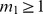, 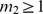 and 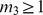, respectively.

1. If 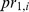 (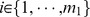) is the unique probe of 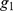 that is related with a phenotype 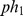, then the gene 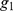 relates with 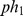 in the same way as 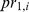. Moreover, the coefficient of the 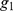-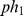 lower logic relationship is equal to the mean uncertainty coefficient of the 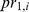-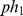 lower logic relationship in both directions.

If 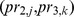 (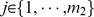 and 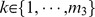) is the unique probe pair related with a phenotype 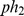, then the gene pair 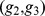 is related with 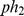 in the same way as the probe pair 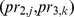. Moreover, the coefficient of the 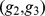-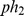 higher logic relationship is the mean uncertainty coefficient of the 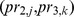-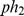 higher logic relationship in both directions.

2. Suppose 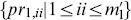 is a probe set of gene 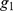, where 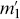 is the size of the set and 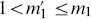. Every probe in the above set is related with a phenotype 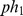 by a lower logic relationship. We define 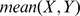 as the mean of 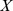 and 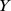, where 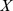 and 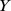 are real numbers. If 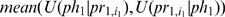 is the largest element in 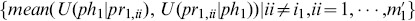, then 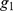 is related with the phenotype 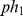 in the same way as the probe 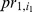, and its coefficient is equal to 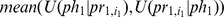.

Similarly, suppose 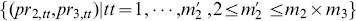 is the probe pair set of gene pairwise 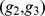, where 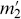 is the size of the set. Every probe pair in the above probe pair set is related with a phenotype 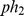 by a higher logic relationship. If 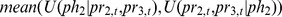 is the maximum mean uncertainty coefficient in 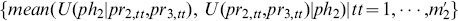, then the gene pair 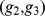 is related with the phenotype 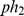 in the same way as the probe pair 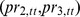, and the coefficient of 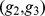-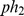 higher logic relationship is equal to 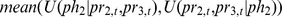.

### Earlier relationship-inference methods

We adapt the two earlier methods suitable for mining gene-phenotype relationships. These methods are described as follows:

1. The non-negative matrix factorization (NMF) method is a model selection method. Given a positive matrix 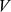 of size 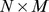, the NMF algorithm iteratively computes an approximation 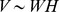, where 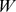 and 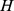 are nonnegative matrics with size 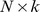 and 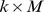, respectively [18]. Each column of 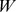 represents a metagene, and the number of columns (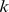) is typically equal to the number of phenotypes. Entry 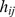 denotes the expression level of metagene 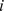 in cluster 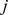. Entry 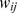 represents the coefficient of gene 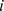 in metagene 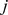. Genes which are more active in the genome have higher coefficient values. When the coefficient values are sorted in descending order, the first one represents the most active gene, while the last one represents the least active. That is, the larger coefficient of a gene in a metagene, the closer relationship between the gene and a phenotype. In this work, we chose the alternate least squares as the algorithm to factorize 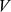 into 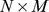 because of the algorithm's speed and robustness. The NMF method is implemented in Matlab using the NMF:DTU toolbox (http://cogsys.imm.dtu.dk/toolbox/nmf/index.html).
2. The relevance analysis (RA) method identifies a potential biological association between a gene and a phenotype by a mutual information value [20]. The mutual information for two discrete random variables 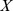 and 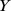 is calculated as:
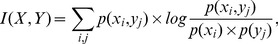
(6)where 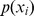 is the probability that 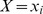, 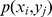 is the joint probability that 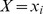 and 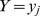, 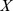 represents a probe profile, and 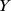 denotes a phenotype profile.

### The classification ability of probes

We evaluate the discriminating ability of probes by constructing a classification model. Given that the competitive neural network (CNN) has produced promising classification accuracy, we apply CNN to build the classification model in this work. Next, we calculate the classification accuracy, which is used as the measure of the probes' classification ability.

The competitive neural network consists of three layers, which are the input layer, the competitive layer and output layer, respectively. An input vector consists of the binary probe data of the evaluated probes in a specimen. During the learning process, for each input vector, the neurons in the competitive layer compete with each other, and the one with the weight vector closest to the input vector is chosen as the winner. The wining neuron is picked up by the output layer, and the output layer classifies the input vector to that class. The classification accuracy is the ratio of the number of specimens which are correctly classified to the total number of specimens.

### Gene ontology analysis

To check how significant the GO term (a pair of GO terms) related with phenotypes, the p-value score and enrichment value are used for gene ontology analysis.

The Gorilla is a web tool to calculate both the p-value score and the enrichment value of a GO term at the top of a ranked list of all genes [46]. We use the Gorilla to compute an exact p-value score and enrichment value for a GO term's significance as follows. Firstly, we rank all the genes by the coefficients of gene-phenotype pairwise combinations. Then, all the gene are uploaded into the Gorilla. Finally, the Gorilla exports the exact p-value score and enrichment value for a GO term's significance.

In addition, we pay attention to the GO terms which are associated with the genes or gene pairs selected. We map the genes (gene pairs) into GO terms and obtain the GO terms (a pair of GO terms) which are related with phenotypes. The p-value score is defined as the probability of obtaining no less number of the same number of gene (genes pairs) by chance by the hypergeometric distribution. It is calculated as follows:
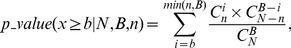
(7)where 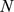 represents the total number of gene (gene pairs), 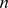 is the number of gene (gene pairs) involved in lower (higher) logic relationships, 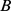 represents the total number of gene (gene pairs) associated with pairs of GO terms, and 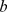 represents the number of the discovered gene (gene pairs) which are associated with the given GO term (a pair of GO terms).

The enrichment value of a GO term (a pair of GO terms) is calculated as follows:
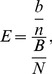
(8)where 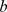, 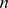, 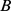 and 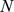 are the same with those in the e.q (7). In the analysis, the significance of a GO term (a pair of GO terms) mainly depends on the p-value scores, as it describes well from a biological point of view.

## Supporting Information

Appendix S1 **Significant GO terms obtained by Gorilla.**
(PDF)

Appendix S2 **The phenotype data and the probe data.**
(ZIP)

Appendix S3 **Matlab codes of the current relationship-inference method.**
(ZIP)

Table S1 **List of probe-AC lower and higher logic relationships identified.**
(PDF)

Table S2 **List of gene-AC lower and higher logic relationships, each of which is generated from more than one probe-AC lower and higher logic relationship.**
(PDF)

Table S3 **List of gene-AC/SCC lower and higher logic relationships identified in this paper.**
(PDF)

Table S4 **Probes sorted by the non-negative matrix factorization method.**
(XLSX)

Table S5 **Two datasets involved the genes which are related with NSCLC.** One dataset includes high frequency genes, and the other contains the genes which are down or up regulated in NSCLC compared to the normal tissue.
(XLSX)

Table S6 **Gene pairs related with AC or SCC through the logic function AND or XOR.**
(PDF)

Table S7 **The genes and probes included in GPL570.**
(ZIP)
