# Supplementary material for: Identification of Logic Relationships between Genes and Subtypes of Non-Small Cell Lung Cancer
Source: PLoS One. 2014 Apr 17;9(4):e94644. doi: 10.1371/journal.pone.0094644 (PMC3990524; doi:10.1371/journal.pone.0094644)
Supplement: Appendix S1 — Significant GO terms obtained by Gorilla. (PDF) [file pone.0094644.s001.pdf]

Supporting Information -Appendix S1: Significant GO terms obtained by Gorilla.

Part A. Significant GO terms on the AC and SCC specimens

| *P-value color scale* | | | | |
|---|---|---|---|---|
| $> 10^{-3}$ | $10^{-3}$ to $10^{-5}$ | $10^{-5}$ to $10^{-7}$ | $10^{-7}$ to $10^{-9}$ | $< 10^{-9}$ |

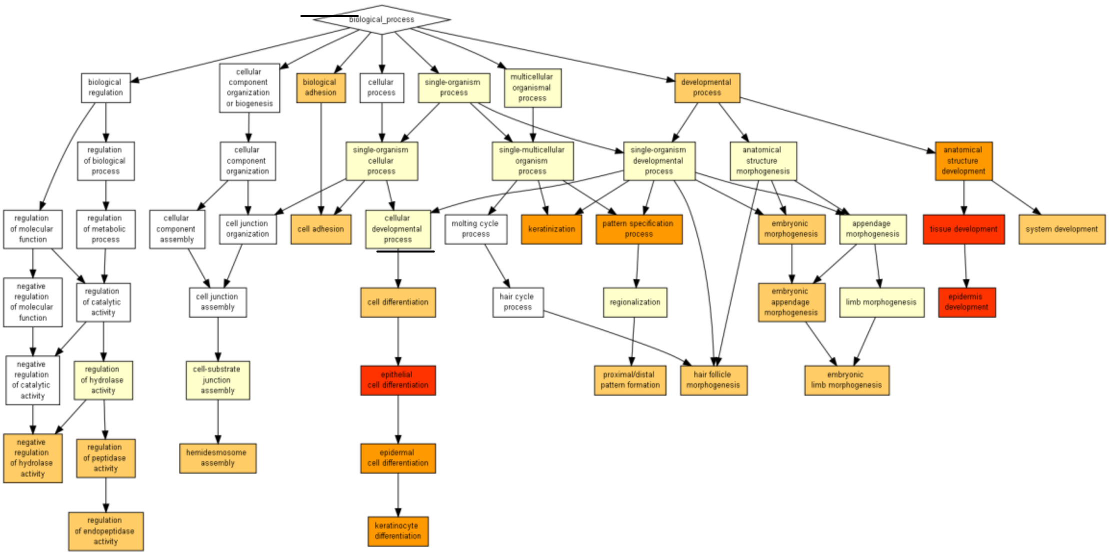


| GO term | Description | P-value | FDR q-value | Enrichment (N, B, n, b) |
|---|---|---|---|---|
| GO:0009888 | tissue development | 6.42E-14 | 7.69E-10 | 7.20 (16095,527,106,25) |
| GO:0008544 | epidermis development | 3.58E-12 | 2.15E-8 | 27.84 (16095,106,60,11) |
| GO:0030855 | epithelial cell differentiation | 7.23E-12 | 2.89E-8 | 5.02 (16095,248,375,29) |
| GO:0031424 | keratinization | 1.24E-9 | 3.73E-6 | 16.90 (16095,25,381,10) |
| GO:0030216 | keratinocyte differentiation | 3.94E-9 | 9.45E-6 | 29.13 (16095,52,85,8) |
| GO:0048856 | anatomical structure development | 5.01E-8 | 1E-4 | 1.68 (16095,2395,484,121) |
| GO:0007389 | pattern specification process | 5.18E-8 | 8.87E-5 | 2.04 (16095,376,1469,70) |
| GO:0009913 | epidermal cell differentiation | 5.97E-8 | 8.94E-5 | 21.04 (16095,72,85,8) |
| GO:0048731 | system development | 7.29E-7 | 9.72E-4 | 2.10 (16095,628,708,58) |
| GO:0032502 | developmental process | 1.58E-6 | 1.89E-3 | 2.23 (16095,3912,70,38) |
| GO:0048598 | embryonic morphogenesis | 1.61E-6 | 1.75E-3 | 3.50 (16095,375,282,23) |

| | | | | |
|---|---|---|---|---|
| GO:0007155 | cell adhesion | 1.97E-6 | 1.96E-3 | 4.45 (16095,759,81,17) |
| GO:0022610 | biological adhesion | 2.05E-6 | 1.89E-3 | 4.44 (16095,761,81,17) |
| GO:0030326 | embryonic limb morphogenesis | 3.75E-6 | 3.21E-3 | 7.39 (16095,85,282,11) |
| GO:0035113 | embryonic appendage morphogenesis | 3.75E-6 | 3E-3 | 7.39 (16095,85,282,11) |
| GO:0052548 | regulation of endopeptidase activity | 4.03E-6 | 3.02E-3 | 5.62 (16095,278,134,13) |
| GO:0030154 | cell differentiation | 4.16E-6 | 2.93E-3 | 3.58 (16095,1364,66,20) |
| GO:0051346 | negative regulation of hydrolase activity | 4.5E-6 | 3E-3 | 1.98 (16095,265,1654,54) |
| GO:0052547 | regulation of peptidase activity | 5.84E-6 | 3.68E-3 | 5.44 (16095,287,134,13) |
| GO:0009954 | proximal/distal pattern formation | 7.84E-6 | 4.7E-3 | 6.63 (16095,26,934,10) |
| GO:0031069 | hair follicle morphogenesis | 7.99E-6 | 4.56E-3 | 17.51 (16095,28,197,6) |
| GO:0031581 | hemidesmosome assembly | 9.77E-6 | 5.33E-3 | 894.17 (16095,12,3,2) |

Species used: Homo sapiens

The system has recognized 18524 genes out of 19803 gene terms entered by the user. 18522 genes were recognized by gene symbol and 2 genes by other gene IDs . 47 duplicate genes were removed (keeping the highest ranking instance of each gene) leaving a total of 18477 genes. Only 16095 of these genes are associated with a GO term.

Part B. Significant GO terms on the NSCLC and Normal specimens

*P-value color scale*

| > $10^{-3}$ | $10^{-3}$ to $10^{-5}$ | $10^{-5}$ to $10^{-7}$ | $10^{-7}$ to $10^{-9}$ | < $10^{-9}$ |
|---|---|---|---|---|

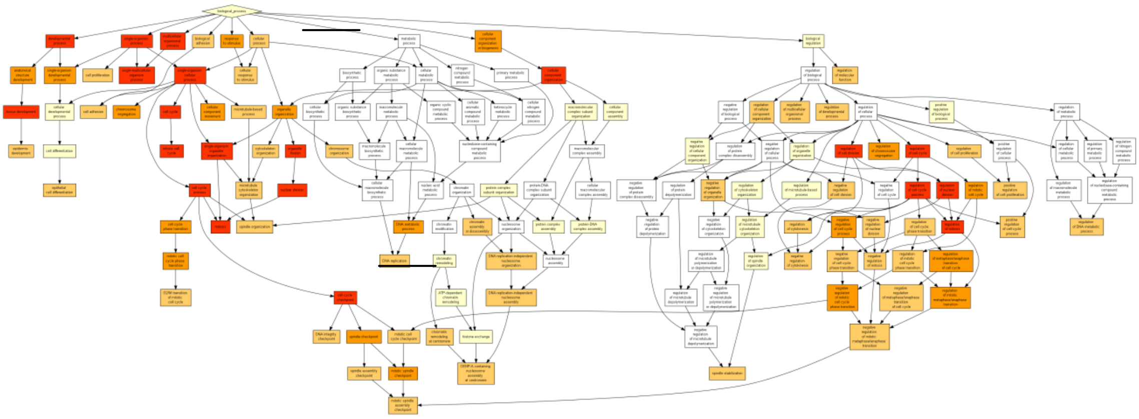

| GO term | Description | P-value | FDR q-value | Enrichment (N, B, n, b) |
|---|---|---|---|---|
| GO:0022402 | cell cycle process | 1.18E-23 | 1.41E-19 | 3.88 (16095,950,349,80) |
| GO:0000278 | mitotic cell cycle | 1.94E-22 | 1.16E-18 | 8.37 (16095,373,201,39) |
| GO:0044763 | single-organism cellular process | 1.03E-21 | 4.13E-18 | 1.41 (16095,8183,596,426) |
| GO:0007049 | cell cycle | 3.16E-21 | 9.47E-18 | 6.42 (16095,545,207,45) |
| GO:0044699 | single-organism process | 1.95E-17 | 4.67E-14 | 1.28 (16095,9830,627,492) |
| GO:0051302 | regulation of cell division | 3.63E-14 | 7.25E-11 | 8.47 (16095,174,273,25) |
| GO:0000280 | nuclear division | 8.36E-14 | 1.43E-10 | 7.08 (16095,229,278,28) |
| GO:0007067 | mitosis | 8.36E-14 | 1.25E-10 | 7.08 (16095,229,278,28) |
| GO:0048285 | organelle fission | 1.53E-13 | 2.03E-10 | 6.64 (16095,253,278,29) |
| GO:0010564 | regulation of cell cycle process | 3.38E-13 | 4.06E-10 | 4.21 (16095,397,376,39) |
| GO:0000075 | cell cycle checkpoint | 4.17E-13 | 4.54E-10 | 6.24 (16095,142,472,26) |
| GO:0007088 | regulation of mitosis | 1.63E-11 | 1.63E-8 | 10.12 (16095,99,273,17) |
| GO:0051783 | regulation of nuclear division | 1.63E-11 | 1.51E-8 | 10.12 (16095,99,273,17) |
| GO:0009888 | tissue development | 5.86E-11 | 5.02E-8 | 1.91 (16095,527,1805,113) |
| GO:0032502 | developmental process | 8.81E-11 | 7.04E-8 | 1.44 (16095,3912,781,274) |
| GO:0051726 | regulation of cell cycle | 4.09E-10 | 3.06E-7 | 2.91 (16095,698,381,48) |
| GO:0032501 | multicellular organismal process | 4.78E-10 | 3.37E-7 | 1.43 (16095,2807,1156,288) |
| GO:0044707 | single-multicellular organism process | 5.21E-10 | 3.47E-7 | 1.43 (16095,2749,1156,283) |

| | | | | |
|---|---|---|---|---|
| GO:0016043 | cellular component organization | 5.66E-10 | 3.57E-7 | 1.66 (16095,3536,398,145) |
| GO:1902589 | single-organism organelle organization | 7.61E-10 | 4.56E-7 | 2.43 (16095,1161,353,62) |
| GO:0071840 | cellular component organization or biogenesis | 1.11E-9 | 6.33E-7 | 1.64 (16095,3567,398,145) |
| GO:1902099 | regulation of metaphase/anaphase transition of cell cycle | 1.19E-9 | 6.49E-7 | 12.63 (16095,41,373,12) |
| GO:0030071 | regulation of mitotic metaphase/anaphase transition | 1.19E-9 | 6.21E-7 | 12.63 (16095,41,373,12) |
| GO:0044772 | mitotic cell cycle phase transition | 2.05E-9 | 1.03E-6 | 6.19 (16095,256,193,19) |
| GO:0031577 | spindle checkpoint | 2.12E-9 | 1.02E-6 | 14.12 (16095,37,339,11) |
| GO:0044770 | cell cycle phase transition | 2.51E-9 | 1.16E-6 | 6.12 (16095,259,193,19) |
| GO:0007346 | regulation of mitotic cell cycle | 3.36E-9 | 1.49E-6 | 4.49 (16095,328,273,25) |
| GO:0044767 | single-organism developmental process | 3.67E-9 | 1.57E-6 | 1.39 (16095,3540,948,290) |
| GO:0010948 | negative regulation of cell cycle process | 4.19E-9 | 1.73E-6 | 5.59 (16095,211,273,20) |
| GO:0006996 | organelle organization | 4.67E-9 | 1.87E-6 | 1.98 (16095,1777,398,87) |
| GO:0071174 | mitotic spindle checkpoint | 1.48E-8 | 5.72E-6 | 13.96 (16095,34,339,10) |
| GO:0007059 | chromosome segregation | 1.78E-8 | 6.66E-6 | 12.62 (16095,83,169,11) |
| GO:0048856 | anatomical structure development | 1.81E-8 | 6.58E-6 | 1.48 (16095,2395,948,209) |
| GO:0050896 | response to stimulus | 3.38E-8 | 1.19E-5 | 1.23 (16095,5753,1197,525) |
| GO:0006928 | cellular component movement | 3.91E-8 | 1.34E-5 | 1.71 (16095,1150,980,120) |
| GO:0006259 | DNA metabolic process | 5.37E-8 | 1.79E-5 | 5.04 (16095,674,90,19) |
| GO:0051983 | regulation of chromosome segregation | 5.59E-8 | 1.81E-5 | 14.54 (16095,27,369,9) |
| GO:1901991 | negative regulation of mitotic cell cycle phase transition | 8.9E-8 | 2.81E-5 | 5.90 (16095,160,273,16) |
| GO:0000226 | microtubule cytoskeleton organization | 1.07E-7 | 3.29E-5 | 3.05 (16095,212,796,32) |
| GO:0007094 | mitotic spindle assembly checkpoint | 1.08E-7 | 3.23E-5 | 13.78 (16095,31,339,9) |
| GO:0045839 | negative regulation of mitosis | 1.11E-7 | 3.24E-5 | 11.58 (16095,41,339,10) |
| GO:0051784 | negative regulation of nuclear division | 1.11E-7 | 3.17E-5 | 11.58 (16095,41,339,10) |
| GO:1901988 | negative regulation of cell cycle phase transition | 1.17E-7 | 3.26E-5 | 5.79 (16095,163,273,16) |

| | | | | |
|---|---|---|---|---|
| GO:0000086 | G2/M transition of mitotic cell cycle | 1.27E-7 | 3.47E-5 | 15.28 (16095,120,79,9) |
| GO:0007093 | mitotic cell cycle checkpoint | 1.31E-7 | 3.5E-5 | 8.96 (16095,79,273,12) |
| GO:0071173 | spindle assembly checkpoint | 1.48E-7 | 3.87E-5 | 13.35 (16095,32,339,9) |
| GO:0042127 | regulation of cell proliferation | 1.64E-7 | 4.19E-5 | 1.80 (16095,1205,704,95) |
| GO:1901990 | regulation of mitotic cell cycle phase transition | 1.9E-7 | 4.73E-5 | 4.94 (16095,215,273,18) |
| GO:0030855 | epithelial cell differentiation | 1.97E-7 | 4.83E-5 | 2.67 (16095,248,924,38) |
| GO:1902100 | negative regulation of metaphase/anaphase transition of cell cycle | 1.98E-7 | 4.75E-5 | 12.95 (16095,33,339,9) |
| GO:0045841 | negative regulation of mitotic metaphase/anaphase transition | 1.98E-7 | 4.66E-5 | 12.95 (16095,33,339,9) |
| GO:1901987 | regulation of cell cycle phase transition | 2.75E-7 | 6.34E-5 | 4.85 (16095,219,273,18) |
| GO:0006333 | chromatin assembly or disassembly | 4.51E-7 | 1.02E-4 | 7.45 (16095,44,589,12) |
| GO:0031055 | chromatin remodeling at centromere | 5.23E-7 | 1.16E-4 | 10.69 (16095,23,589,9) |
| GO:0007010 | cytoskeleton organization | 5.34E-7 | 1.16E-4 | 2.12 (16095,545,807,58) |
| GO:0051052 | regulation of DNA metabolic process | 5.96E-7 | 1.28E-4 | 4.87 (16095,234,240,17) |
| GO:0007017 | microtubule-based process | 6.56E-7 | 1.38E-4 | 2.89 (16095,377,472,32) |
| GO:0051128 | regulation of cellular component organization | 7.22E-7 | 1.49E-4 | 1.46 (16095,1399,1449,184) |
| GO:0006260 | DNA replication | 8.89E-7 | 1.81E-4 | 6.97 (16095,169,164,12) |
| GO:0008283 | cell proliferation | 9.86E-7 | 1.97E-4 | 2.23 (16095,576,627,50) |
| GO:0009987 | cellular process | 1.37E-6 | 2.69E-4 | 1.16 (16095,11818,365,310) |
| GO:0007051 | spindle organization | 1.45E-6 | 2.8E-4 | 5.75 (16095,69,568,14) |
| GO:0034724 | DNA replication-independent nucleosome organization | 1.75E-6 | 3.34E-4 | 6.25 (16095,21,1348,11) |
| GO:0034080 | CENP-A containing nucleosome assembly at centromere | 1.75E-6 | 3.29E-4 | 6.25 (16095,21,1348,11) |
| GO:0006336 | DNA replication-independent nucleosome assembly | 1.75E-6 | 3.24E-4 | 6.25 (16095,21,1348,11) |
| GO:0090068 | positive regulation of cell cycle process | 1.78E-6 | 3.23E-4 | 6.59 (16095,188,156,12) |

| | | | | |
|---|---|---|---|---|
| GO:0010639 | negative regulation of organelle organization | 1.9E-6 | 3.4E-4 | 3.99 (16095,188,408,19) |
| GO:0008544 | epidermis development | 2.28E-6 | 4.02E-4 | 2.84 (16095,106,1549,29) |
| GO:0043146 | spindle stabilization | 3.18E-6 | 5.53E-4 | 83.39 (16095,3,193,3) |
| GO:0031570 | DNA integrity checkpoint | 4.17E-6 | 7.15E-4 | 5.81 (16095,79,456,13) |
| GO:0032465 | regulation of cytokinesis | 4.6E-6 | 7.77E-4 | 13.96 (16095,30,269,7) |
| GO:0051782 | negative regulation of cell division | 4.94E-6 | 8.23E-4 | 22.06 (16095,8,456,5) |
| GO:0065009 | regulation of molecular function | 5.25E-6 | 8.62E-4 | 6.55 (16095,2010,11,9) |
| GO:0032466 | negative regulation of cytokinesis | 5.58E-6 | 9.05E-4 | 69.98 (16095,3,230,3) |
| GO:0007155 | cell adhesion | 6.63E-6 | 1.06E-3 | 1.51 (16095,759,1820,130) |
| GO:0008284 | positive regulation of cell proliferation | 6.78E-6 | 1.07E-3 | 2.38 (16095,671,383,38) |
| GO:0051716 | cellular response to stimulus | 6.98E-6 | 1.09E-3 | 1.18 (16095,4524,1802,598) |
| GO:0051239 | regulation of multicellular organismal process | 7.05E-6 | 1.08E-3 | 1.31 (16095,1857,1837,278) |
| GO:0022610 | biological adhesion | 7.97E-6 | 1.21E-3 | 1.51 (16095,761,1820,130) |
| GO:0050793 | regulation of developmental process | 8.77E-6 | 1.31E-3 | 1.34 (16095,1562,1837,239) |
| GO:0051276 | chromosome organization | 9.7E-6 | 1.44E-3 | 4.49 (16095,640,84,15) |

Species used: Homo sapiens

The system has recognized 18524 genes out of 19804 gene terms entered by the user. 18522 genes were recognized by gene symbol and 2 genes by other gene IDs . 47 duplicate genes were removed (keeping the highest ranking instance of each gene) leaving a total of 18477 genes. Only 16095 of these genes are associated with a GO term.

'P-value' is the enrichment p-value computed according to the mHG or HG model. This p-value is not corrected for multiple testing of 11988 GO terms. 'FDR q-value' is the correction of the above p-value for multiple testing using the Benjamini and Hochberg (1995) method.
Namely, for the ith term (ranked according to p-value) the FDR q-value is (p-value * number of GO terms) / i.
Enrichment (N, B, n, b) is defined as follows:
N - is the total number of genes
B - is the total number of genes associated with a specific GO term
n - is the number of genes in the top of the user's input list or in the target set when appropriate
b - is the number of genes in the intersection
Enrichment = (b/n) / (B/N).
