# Supplementary material for: Identification of Logic Relationships between Genes and Subtypes of Non-Small Cell Lung Cancer
Source: PLoS One. 2014 Apr 17;9(4):e94644. doi: 10.1371/journal.pone.0094644 (PMC3990524; doi:10.1371/journal.pone.0094644)
Supplement: Table S1 — List of probe-AC lower and higher logic relationships identified. (PDF) [file pone.0094644.s004.pdf]

Supporting Information -Table S1: List of probe-AC lower and higher logic relationships identified in this paper.

Table A 274 probe-AC lower logic relationships

Probe	Probe-subtype coefficient	Subtype-probe coefficient	Type
'1552487_a_at'	0.61	0.61	2
'1552543_a_at'	0.22	0.25	2
'1552834_at'	0.28	0.33	1
'1553158_at'	0.25	0.25	2
'1553169_at'	0.21	0.26	1
'1553171_x_at'	0.28	0.28	1
'1553295_at'	0.29	0.29	2
'1553705_a_at'	0.33	0.34	2
'1553938_a_at'	0.36	0.36	1
'1554050_at'	0.31	0.31	1
'1554246_at'	0.33	0.34	1
'1554252_a_at'	0.49	0.51	2
'1554253_a_at'	0.24	0.24	2
'1554586_a_at'	0.20	0.22	1
'1555203_s_at'	0.23	0.23	1
'1555581_a_at'	0.31	0.38	2
'1555942_a_at'	0.31	0.35	2
'1555962_at'	0.24	0.25	1
'1555993_at'	0.18	0.23	1
'1556012_at'	0.26	0.27	1
'1556793_a_at'	0.38	0.40	2
'1557918_s_at'	0.38	0.40	2
'1558815_at'	0.20	0.22	1
'1559606_at'	0.38	0.38	2
'1559607_s_at'	0.43	0.44	2
'1559633_a_at'	0.32	0.32	2
'1560407_at'	0.26	0.29	2
'1563805_a_at'	0.18	0.25	2
'1563900_at'	0.42	0.43	2
'1564307_a_at'	0.40	0.41	2
'1565936_a_at'	0.30	0.31	1
'1565937_a_at'	0.24	0.29	1
'1566140_at'	0.34	0.38	1
'1569203_at'	0.24	0.24	1
'1569688_at'	0.19	0.25	1
'201249_at'	0.31	0.31	2
'201710_at'	0.19	0.23	2

'201820_at'	0.68	0.68	2
'202094_at'	0.23	0.31	2
'202235_at'	0.23	0.25	2
'202504_at'	0.29	0.35	2
'202755_s_at'	0.35	0.35	2
'203365_s_at'	0.30	0.32	1
'203691_at'	0.32	0.34	2
'203716_s_at'	0.22	0.23	1
'203797_at'	0.24	0.28	2
'203953_s_at'	0.35	0.37	1
'204603_at'	0.19	0.28	2
'204614_at'	0.43	0.43	2
'204855_at'	0.31	0.35	2
'204948_s_at'	0.20	0.25	2
'204952_at'	0.33	0.33	2
'205064_at'	0.30	0.32	2
'205102_at'	0.25	0.29	1
'205109_s_at'	0.29	0.31	2
'205186_at'	0.33	0.34	1
'205216_s_at'	0.30	0.31	1
'205309_at'	0.49	0.50	1
'205313_at'	0.40	0.40	1
'205432_at'	0.19	0.24	1
'205490_x_at'	0.33	0.33	2
'205595_at'	0.66	0.66	2
'205614_x_at'	0.21	0.23	1
'205637_s_at'	0.31	0.32	2
'205640_at'	0.25	0.27	1
'205646_s_at'	0.20	0.28	2
'205724_at'	0.57	0.57	2
'205894_at'	0.26	0.26	1
'205916_at'	0.35	0.35	2
'205942_s_at'	0.25	0.25	1
'206008_at'	0.18	0.28	2
'206032_at'	0.35	0.38	2
'206033_s_at'	0.52	0.52	2
'206046_at'	0.54	0.55	2
'206122_at'	0.43	0.44	2
'206156_at'	0.66	0.66	2
'206164_at'	0.59	0.59	2
'206165_s_at'	0.38	0.39	2
'206166_s_at'	0.68	0.68	2
'206307_s_at'	0.37	0.37	2

'206458_s_at'	0.33	0.35	2
'206459_s_at'	0.19	0.22	2
'206581_at'	0.43	0.43	2
'206677_at'	0.27	0.30	2
'206751_s_at'	0.19	0.28	2
'206912_at'	0.24	0.26	2
'207206_s_at'	0.27	0.27	2
'207345_at'	0.19	0.24	2
'207382_at'	0.51	0.51	2
'207397_s_at'	0.20	0.28	2
'207455_at'	0.19	0.22	2
'207602_at'	0.38	0.42	2
'207675_x_at'	0.22	0.28	2
'207837_at'	0.25	0.25	1
'207935_s_at'	0.42	0.43	2
'207949_s_at'	0.24	0.29	1
'208153_s_at'	0.54	0.55	2
'208209_s_at'	0.19	0.26	1
'208502_s_at'	0.27	0.29	2
'208539_x_at'	0.37	0.37	2
'208600_s_at'	0.28	0.32	1
'209125_at'	0.28	0.33	2
'209126_x_at'	0.55	0.56	2
'209351_at'	0.38	0.38	2
'209504_s_at'	0.28	0.31	1
'209587_at'	0.29	0.31	2
'209590_at'	0.33	0.33	2
'209591_s_at'	0.32	0.32	2
'209719_x_at'	0.18	0.22	2
'209720_s_at'	0.27	0.29	2
'209800_at'	0.41	0.41	2
'210020_x_at'	0.26	0.27	2
'210237_at'	0.29	0.33	2
'210505_at'	0.40	0.40	2
'210521_s_at'	0.27	0.35	2
'210673_x_at'	0.51	0.51	1
'210721_s_at'	0.28	0.33	2
'210945_at'	0.31	0.33	2
'211002_s_at'	0.59	0.59	2
'211194_s_at'	0.51	0.51	2
'211195_s_at'	0.27	0.36	2
'211259_s_at'	0.21	0.24	2
'211361_s_at'	0.63	0.63	2

'211362_s_at'	0.40	0.40	2
'211401_s_at'	0.29	0.29	2
'211473_s_at'	0.20	0.30	2
'211834_s_at'	0.42	0.43	2
'211906_s_at'	0.28	0.28	2
'212478_at'	0.25	0.25	2
'213308_at'	0.21	0.25	1
'213441_x_at'	0.23	0.25	1
'213533_at'	0.18	0.23	2
'213680_at'	0.46	0.47	2
'213707_s_at'	0.36	0.36	2
'213722_at'	0.26	0.28	2
'213796_at'	0.38	0.38	2
'213820_s_at'	0.32	0.32	2
'213990_s_at'	0.22	0.31	2
'213992_at'	0.38	0.38	2
'214549_x_at'	0.31	0.32	2
'214595_at'	0.23	0.27	2
'214596_at'	0.33	0.33	2
'214639_s_at'	0.25	0.26	2
'214641_at'	0.29	0.34	1
'214680_at'	0.35	0.41	2
'214846_s_at'	0.27	0.29	1
'215300_s_at'	0.44	0.45	1
'215559_at'	0.29	0.34	1
'215563_s_at'	0.28	0.33	1
'215724_at'	0.25	0.31	2
'216258_s_at'	0.50	0.52	2
'216488_s_at'	0.42	0.42	1
'216705_s_at'	0.21	0.23	2
'216918_s_at'	0.69	0.69	2
'217040_x_at'	0.20	0.28	2
'217272_s_at'	0.57	0.57	2
'217305_s_at'	0.19	0.24	2
'217312_s_at'	0.45	0.45	2
'217528_at'	0.60	0.60	2
'218553_s_at'	0.20	0.23	2
'218629_at'	0.24	0.25	2
'218657_at'	0.28	0.33	2
'218832_x_at'	0.30	0.31	1
'218931_at'	0.26	0.26	1
'218990_s_at'	0.31	0.31	2
'219227_at'	0.24	0.24	1

'219498_s_at'	0.25	0.27	2
'219511_s_at'	0.21	0.22	2
'219536_s_at'	0.29	0.31	2
'219545_at'	0.24	0.27	1
'219554_at'	0.24	0.28	2
'219855_at'	0.33	0.35	2
'219936_s_at'	0.22	0.24	2
'220009_at'	0.26	0.28	1
'220026_at'	0.26	0.31	2
'220102_at'	0.30	0.34	2
'220108_at'	0.25	0.28	1
'220198_s_at'	0.30	0.30	2
'220296_at'	0.22	0.27	1
'220393_at'	0.31	0.31	1
'220559_at'	0.22	0.24	2
'220622_at'	0.27	0.33	1
'220751_s_at'	0.22	0.26	1
'220816_at'	0.33	0.33	2
'221291_at'	0.38	0.39	2
'221779_at'	0.27	0.28	2
'221795_at'	0.39	0.39	2
'221796_at'	0.23	0.26	2
'221854_at'	0.49	0.49	2
'222678_s_at'	0.18	0.26	2
'222892_s_at'	0.40	0.41	2
'222938_x_at'	0.23	0.23	1
'223232_s_at'	0.19	0.24	1
'223694_at'	0.29	0.29	2
'223832_s_at'	0.34	0.35	2
'224204_x_at'	0.27	0.30	2
'224209_s_at'	0.26	0.28	2
'224328_s_at'	0.25	0.29	2
'224394_at'	0.20	0.27	2
'224397_s_at'	0.20	0.26	2
'224458_at'	0.23	0.29	2
'225211_at'	0.49	0.49	2
'225531_at'	0.19	0.21	1
'225822_at'	0.20	0.28	1
'226553_at'	0.28	0.28	1
'226570_at'	0.27	0.29	2
'226755_at'	0.57	0.57	2
'226891_at'	0.25	0.28	2
'226973_at'	0.37	0.37	1

'227226_at'	0.33	0.33	2
'227282_at'	0.33	0.33	2
'227429_at'	0.27	0.27	1
'227735_s_at'	0.32	0.36	2
'227736_at'	0.34	0.38	2
'228375_at'	0.37	0.38	2
'228806_at'	0.50	0.50	1
'228877_at'	0.46	0.49	1
'228969_at'	0.21	0.24	1
'229030_at'	0.19	0.26	1
'229105_at'	0.37	0.41	1
'229178_at'	0.27	0.28	1
'229245_at'	0.37	0.39	1
'229290_at'	0.48	0.48	2
'229372_at'	0.53	0.53	1
'229385_s_at'	0.20	0.20	2
'229463_at'	0.27	0.34	2
'229599_at'	0.25	0.25	1
'229761_at'	0.23	0.23	2
'230021_at'	0.21	0.21	2
'230087_at'	0.24	0.24	2
'230134_s_at'	0.23	0.23	2
'230398_at'	0.28	0.28	2
'230464_at'	0.61	0.62	2
'230835_at'	0.22	0.32	2
'231070_at'	0.19	0.25	1
'231430_at'	0.29	0.33	2
'231771_at'	0.40	0.41	2
'231800_s_at'	0.25	0.26	2
'231867_at'	0.42	0.42	2
'231928_at'	0.36	0.37	2
'232082_x_at'	0.39	0.39	2
'232116_at'	0.36	0.36	2
'232263_at'	0.26	0.28	2
'232553_at'	0.24	0.30	2
'232602_at'	0.25	0.27	1
'232765_x_at'	0.32	0.34	1
'233294_at'	0.24	0.27	2
'233801_s_at'	0.19	0.29	2
'233882_s_at'	0.26	0.29	2
'234700_s_at'	0.28	0.36	2
'235075_at'	0.61	0.61	2
'235269_at'	0.28	0.28	2

'235272_at'	0.23	0.26	2
'235795_at'	0.36	0.37	2
'235852_at'	0.20	0.23	2
'236083_at'	0.19	0.29	1
'236095_at'	0.44	0.46	2
'236098_at'	0.20	0.25	1
'236681_at'	0.24	0.26	2
'236741_at'	0.26	0.27	2
'236979_at'	0.28	0.29	1
'238419_at'	0.28	0.28	2
'238811_at'	0.18	0.25	2
'239309_at'	0.27	0.27	2
'240353_s_at'	0.33	0.35	2
'240354_at'	0.18	0.22	2
'241459_at'	0.26	0.26	1
'242138_at'	0.30	0.34	2
'242204_at'	0.27	0.27	2
'242940_x_at'	0.24	0.25	2
'243623_at'	0.18	0.28	1
'243681_at'	0.26	0.26	1
'244056_at'	0.20	0.27	1
'244463_at'	0.56	0.56	2

Table B 170 probe-AC higher logic relationships

Probe1	Probe2	Coefficient (probe1,probe2-subtype)	Coefficient (subtype-probe1,probe2)	Type
'1552478_a_at'	'1552496_a_at'	0.30	0.31	6_2
'1552478_a_at'	'208608_s_at'	0.33	0.35	6_2
'1552478_a_at'	'210738_s_at'	0.33	0.34	6_2
'1552478_a_at'	'214708_at'	0.33	0.36	6_2
'1552478_a_at'	'234305_s_at'	0.31	0.31	2
'1552478_a_at'	'235248_at'	0.31	0.33	6_2
'1552496_a_at'	'1554835_a_at'	0.30	0.30	6_1
'1552496_a_at'	'205348_s_at'	0.31	0.31	6_1
'1552496_a_at'	'205701_at'	0.30	0.31	6_1
'1552496_a_at'	'244321_at'	0.34	0.34	6_1
'1552665_at'	'1555772_a_at'	0.32	0.34	6_1
'1552665_at'	'206941_x_at'	0.31	0.35	3
'1553183_at'	'203438_at'	0.31	0.31	6_1
'1553183_at'	'230682_x_at'	0.31	0.32	3

'1553423_a_at'	'235486_at'	0.31	0.31	5_1
'1553454_at'	'214254_at'	0.31	0.31	4
'1553604_at'	'214708_at'	0.30	0.32	6_2
'1553654_at'	'243021_at'	0.30	0.31	5_2
'1554062_at'	'205506_at'	0.31	0.31	6_2
'1554062_at'	'228912_at'	0.31	0.32	6_2
'1554835_a_at'	'202756_s_at'	0.33	0.33	2
'1554835_a_at'	'206290_s_at'	0.33	0.33	6_2
'1554835_a_at'	'234305_s_at'	0.30	0.30	2
'1554912_at'	'227578_at'	0.30	0.36	5_1
'1554960_at'	'235486_at'	0.30	0.31	4
'1555006_at'	'1557675_at'	0.30	0.31	5_2
'1555006_at'	'1563498_s_at'	0.32	0.33	5_2
'1555006_at'	'220414_at'	0.31	0.32	4
'1555383_a_at'	'235486_at'	0.31	0.32	4
'1556476_at'	'206010_at'	0.30	0.31	5_2
'1557474_at'	'205649_s_at'	0.31	0.32	3
'1558523_at'	'228463_at'	0.31	0.32	3
'1562697_at'	'244321_at'	0.32	0.32	6_1
'1569886_a_at'	'205506_at'	0.33	0.33	3
'1569886_a_at'	'205649_s_at'	0.31	0.32	3
'1569886_a_at'	'228463_at'	0.33	0.34	3
'1570032_at'	'209277_at'	0.31	0.31	5_2
'1570032_at'	'230863_at'	0.31	0.31	5_2
'1570032_at'	'238962_at'	0.31	0.31	5_2
'201474_s_at'	'223779_at'	0.30	0.30	1
'202756_s_at'	'214486_x_at'	0.33	0.33	6_2
'202756_s_at'	'230682_x_at'	0.31	0.32	6_2
'202756_s_at'	'235287_at'	0.31	0.31	2
'202831_at'	'204973_at'	0.33	0.33	6_2
'202831_at'	'205649_s_at'	0.36	0.36	6_2
'202831_at'	'205650_s_at'	0.34	0.34	6_2
'202831_at'	'211488_s_at'	0.31	0.31	2
'202831_at'	'222712_s_at'	0.31	0.31	6_2
'202831_at'	'228463_at'	0.33	0.33	6_2
'202831_at'	'232109_at'	0.31	0.31	6_2
'202831_at'	'244353_s_at'	0.32	0.32	2
'203438_at'	'205649_s_at'	0.31	0.31	6_2
'203438_at'	'205650_s_at'	0.30	0.31	6_2
'203438_at'	'209441_at'	0.32	0.33	6_2
'203438_at'	'224476_s_at'	0.30	0.38	6_1
'203798_s_at'	'205649_s_at'	0.31	0.32	6_2
'203798_s_at'	'214486_x_at'	0.30	0.30	6_2

'203798_s_at'	'214708_at'	0.35	0.36	6_2
'203798_s_at'	'225130_at'	0.34	0.34	6_2
'203798_s_at'	'230349_at'	0.30	0.32	6_2
'203798_s_at'	'239178_at'	0.30	0.30	6_2
'204471_at'	'219727_at'	0.32	0.35	2
'204492_at'	'205506_at'	0.30	0.30	6_2
'204492_at'	'228912_at'	0.33	0.33	6_2
'204654_s_at'	'209699_x_at'	0.31	0.31	2
'204654_s_at'	'210347_s_at'	0.31	0.32	2
'204654_s_at'	'219998_at'	0.31	0.31	2
'204654_s_at'	'224555_x_at'	0.30	0.30	6_2
'204654_s_at'	'230682_x_at'	0.34	0.34	6_2
'204934_s_at'	'214254_at'	0.30	0.31	5_1
'204973_at'	'209699_x_at'	0.33	0.33	6_1
'204973_at'	'219727_at'	0.31	0.31	6_1
'204989_s_at'	'229400_at'	0.30	0.31	2
'204989_s_at'	'230682_x_at'	0.32	0.34	6_2
'205085_at'	'219727_at'	0.31	0.31	2
'205157_s_at'	'232109_at'	0.32	0.33	6_2
'205253_at'	'219926_at'	0.30	0.31	4
'205253_at'	'220322_at'	0.31	0.32	4
'205253_at'	'224329_s_at'	0.30	0.30	4
'205253_at'	'235486_at'	0.32	0.34	4
'205268_s_at'	'220414_at'	0.30	0.31	4
'205348_s_at'	'205649_s_at'	0.31	0.31	6_2
'205388_at'	'205650_s_at'	0.31	0.31	3
'205388_at'	'206290_s_at'	0.32	0.32	3
'205388_at'	'233720_at'	0.31	0.32	3
'205388_at'	'235486_at'	0.30	0.31	6_1
'205506_at'	'216898_s_at'	0.30	0.30	3
'205649_s_at'	'209699_x_at'	0.30	0.31	6_1
'205649_s_at'	'214240_at'	0.31	0.31	6_1
'205649_s_at'	'230682_x_at'	0.33	0.34	3
'206290_s_at'	'214486_x_at'	0.30	0.30	3
'206290_s_at'	'230682_x_at'	0.32	0.33	3
'206300_s_at'	'206859_s_at'	0.32	0.32	6_2
'206300_s_at'	'208106_x_at'	0.30	0.30	6_2
'206300_s_at'	'210728_s_at'	0.33	0.35	7
'206300_s_at'	'214708_at'	0.31	0.31	6_2
'206300_s_at'	'217495_x_at'	0.31	0.33	7
'206300_s_at'	'219727_at'	0.33	0.33	2
'206300_s_at'	'224156_x_at'	0.32	0.33	6_2
'206300_s_at'	'225130_at'	0.30	0.30	6_2

'206300_s_at'	'228463_at'	0.36	0.36	6_2
'206300_s_at'	'228625_at'	0.31	0.31	2
'206300_s_at'	'228912_at'	0.33	0.33	6_2
'206343_s_at'	'214774_x_at'	0.30	0.31	5_2
'206343_s_at'	'215108_x_at'	0.30	0.31	5_2
'206343_s_at'	'216623_x_at'	0.31	0.31	5_2
'206343_s_at'	'229177_at'	0.30	0.30	5_2
'206343_s_at'	'243021_at'	0.32	0.33	5_2
'206505_at'	'230682_x_at'	0.33	0.33	3
'206515_at'	'206941_x_at'	0.31	0.32	6_2
'206941_x_at'	'219727_at'	0.32	0.33	6_1
'206941_x_at'	'244321_at'	0.31	0.31	6_1
'207307_at'	'235486_at'	0.30	0.31	4
'209441_at'	'225094_at'	0.30	0.31	6_1
'209618_at'	'230682_x_at'	0.31	0.31	3
'209985_s_at'	'230682_x_at'	0.32	0.32	3
'210008_s_at'	'228802_at'	0.30	0.31	6_2
'210103_s_at'	'219727_at'	0.31	0.31	6_1
'210192_at'	'224555_x_at'	0.30	0.30	3
'210272_at'	'214254_at'	0.31	0.31	5_1
'210272_at'	'220262_s_at'	0.32	0.32	5_1
'210727_at'	'230682_x_at'	0.36	0.36	3
'210728_s_at'	'230682_x_at'	0.33	0.33	3
'210906_x_at'	'233198_at'	0.30	0.30	1
'211121_s_at'	'235486_at'	0.30	0.31	5_1
'212823_s_at'	'230822_at'	0.34	0.35	6_2
'212823_s_at'	'235287_at'	0.31	0.31	2
'212823_s_at'	'238878_at'	0.32	0.32	6_2
'212823_s_at'	'240282_at'	0.31	0.33	6_2
'213432_at'	'243116_at'	0.32	0.32	3
'213768_s_at'	'230682_x_at'	0.32	0.32	3
'214404_x_at'	'218445_at'	0.30	0.30	6_1
'214404_x_at'	'223468_s_at'	0.30	0.32	6_1
'214486_x_at'	'219727_at'	0.31	0.31	6_1
'214486_x_at'	'223950_s_at'	0.32	0.34	6_1
'214486_x_at'	'226638_at'	0.31	0.32	6_1
'214486_x_at'	'228912_at'	0.30	0.30	3
'214602_at'	'219727_at'	0.31	0.31	6_1
'214898_x_at'	'227759_at'	0.31	0.32	6_1
'216850_at'	'219301_s_at'	0.33	0.33	6_1
'216850_at'	'219727_at'	0.31	0.31	6_1
'216850_at'	'228912_at'	0.31	0.32	3
'216850_at'	'238878_at'	0.31	0.31	3

'217495_x_at'	'230682_x_at'	0.31	0.31	3
'218413_s_at'	'223779_at'	0.30	0.31	5_2
'218445_at'	'244321_at'	0.30	0.30	2
'219558_at'	'219727_at'	0.32	0.32	2
'219558_at'	'228912_at'	0.30	0.30	6_2
'219727_at'	'233720_at'	0.35	0.37	6_2
'219727_at'	'239719_at'	0.33	0.33	2
'219926_at'	'220262_s_at'	0.34	0.34	4
'220709_at'	'221052_at'	0.31	0.31	5_2
'221967_at'	'228463_at'	0.31	0.31	6_2
'222712_s_at'	'230822_at'	0.31	0.32	3
'223169_s_at'	'235486_at'	0.31	0.31	5_1
'223642_at'	'224555_x_at'	0.33	0.33	6_2
'224555_x_at'	'244321_at'	0.33	0.33	6_1
'227806_at'	'229073_at'	0.30	0.31	5_2
'227865_at'	'235486_at'	0.30	0.30	5_1
'228342_s_at'	'236436_at'	0.30	0.30	3
'228342_s_at'	'244321_at'	0.32	0.32	6_1
'228463_at'	'230682_x_at'	0.33	0.35	3
'228625_at'	'244321_at'	0.31	0.31	2
'228912_at'	'230682_x_at'	0.31	0.32	3
'229407_at'	'233720_at'	0.31	0.32	6_2
'230682_x_at'	'231179_at'	0.31	0.31	3
'233720_at'	'238878_at'	0.33	0.34	3
'236055_at'	'238962_at'	0.30	0.31	5_2
'238176_at'	'244321_at'	0.33	0.33	6_1
'238878_at'	'239719_at'	0.30	0.30	6_1
