# Supplementary material for: Identification of Logic Relationships between Genes and Subtypes of Non-Small Cell Lung Cancer
Source: PLoS One. 2014 Apr 17;9(4):e94644. doi: 10.1371/journal.pone.0094644 (PMC3990524; doi:10.1371/journal.pone.0094644)
Supplement: Table S2 — List of gene-AC lower and higher logic relationships, each of which is generated from more than one probe-AC lower and higher logic relationship. (PDF) [file pone.0094644.s005.pdf]

Supporting Information -Table S2: List of gene-AC lower and higher logic relationships, each of which is generated from more than one probe-AC lower and higher logic relationship.

Table A 44 gene-AC lower logic relationships from 101 probe-AC lower logic relationships

Probe	Gene	Type (Gene-AC)	Type (Gene-SCC)
'206046_at'	'ADAM23'	2	1
'244463_at'	'ADAM23'	2	1
'207675_x_at'	'ARTN'	2	1
'210237_at'	'ARTN'	2	1
'236083_at'	'BCL2L15'	1	2
'236979_at'	'BCL2L15'	1	2
'209590_at'	'BMP7'	2	1
'209591_s_at'	'BMP7'	2	1
'211259_s_at'	'BMP7'	2	1
'1552487_a_at'	'BNC1'	2	1
'206581_at'	'BNC1'	2	1
'227735_s_at'	'C10orf99'	2	1
'227736_at'	'C10orf99'	2	1
'240353_s_at'	'C12orf54'	2	1
'240354_at'	'C12orf54'	2	1
'1553705_a_at'	'CHRM3'	2	1
'1559633_a_at'	'CHRM3'	2	1
'214596_at'	'CHRM3'	2	1
'206164_at'	'CLCA2'	2	1
'206165_s_at'	'CLCA2'	2	1
'206166_s_at'	'CLCA2'	2	1
'217528_at'	'CLCA2'	2	1
'210945_at'	'COL4A6'	2	1
'211473_s_at'	'COL4A6'	2	1
'213992_at'	'COL4A6'	2	1
'239309_at'	'DLX6'	2	1
'242940_x_at'	'DLX6'	2	1
'206032_at'	'DSC3'	2	1
'206033_s_at'	'DSC3'	2	1
'205595_at'	'DSG3'	2	1
'235075_at'	'DSG3'	2	1
'1556793_a_at'	'FAM83C'	2	1
'1563805_a_at'	'FAM83C'	2	1
'1569688_at'	'FMO5'	1	2
'215300_s_at'	'FMO5'	1	2
'204948_s_at'	'FST'	2	1
'207345_at'	'FST'	2	1

'1559606_at'	'GBP6'	2	1
'1559607_s_at'	'GBP6'	2	1
'208600_s_at'	'GPR39'	1	2
'229105_at'	'GPR39'	1	2
'207397_s_at'	'HOXD13'	2	1
'236681_at'	'HOXD13'	2	1
'209126_x_at'	'KRT6B'	2	1
'213680_at'	'KRT6B'	2	1
'1554252_a_at'	'LASS3'	2	1
'1554253_a_at'	'LASS3'	2	1
'1565936_a_at'	'LMO3'	1	2
'1565937_a_at'	'LMO3'	1	2
'1555942_a_at'	'LOC642587'	2	1
'226755_at'	'LOC642587'	2	1
'1553169_at'	'LRRN4'	1	2
'1553171_x_at'	'LRRN4'	1	2
'214680_at'	'NTRK2'	2	1
'221795_at'	'NTRK2'	2	1
'221796_at'	'NTRK2'	2	1
'229463_at'	'NTRK2'	2	1
'236095_at'	'NTRK2'	2	1
'210721_s_at'	'PAK7'	2	1
'213990_s_at'	'PAK7'	2	1
'205646_s_at'	'PAX6'	2	1
'235795_at'	'PAX6'	2	1
'206751_s_at'	'PCYT1B'	2	1
'232553_at'	'PCYT1B'	2	1
'208502_s_at'	'PITX1'	2	1
'209587_at'	'PITX1'	2	1
'205724_at'	'PKP1'	2	1
'221854_at'	'PKP1'	2	1
'233801_s_at'	'SEMA6D'	2	1
'233882_s_at'	'SEMA6D'	2	1
'211361_s_at'	'SERPINB13'	2	1
'211362_s_at'	'SERPINB13'	2	1
'216258_s_at'	'SERPINB13'	2	1
'217272_s_at'	'SERPINB13'	2	1
'209719_x_at'	'SERPINB3'	2	1
'209720_s_at'	'SERPINB3'	2	1
'213308_at'	'SHANK2'	1	2
'243681_at'	'SHANK2'	1	2
'1557918_s_at'	'SLC16A1'	2	1
'202235_at'	'SLC16A1'	2	1

'1554050_at'	'SMPDL3B'	1	2
'205309_at'	'SMPDL3B'	1	2
'206122_at'	'SOX15'	2	1
'217040_x_at'	'SOX15'	2	1
'213796_at'	'SPRR1A'	2	1
'214549_x_at'	'SPRR1A'	2	1
'218990_s_at'	'SPRR3'	2	1
'232082_x_at'	'SPRR3'	2	1
'1552543_a_at'	'STON2'	2	1
'235852_at'	'STON2'	2	1
'205102_at'	'TMPRSS2'	1	2
'226553_at'	'TMPRSS2'	1	2
'1555581_a_at'	'TP63'	2	1
'207382_at'	'TP63'	2	1
'211194_s_at'	'TP63'	2	1
'211195_s_at'	'TP63'	2	1
'211834_s_at'	'TP63'	2	1
'202504_at'	'TRIM29'	2	1
'211002_s_at'	'TRIM29'	2	1
'206458_s_at'	'WNT2B'	2	1
'206459_s_at'	'WNT2B'	2	1

Table B Six gene-AC higher logic relationships from 14 probe-AC higher logic relationships

Probe1	Probe2	Gene1	Gene2	Type (Gene-AC)	Type (Gene-SCC)
'209985_s_at'	'230682_x_at'	'ASCL1'	'ABCC3'	3	4
'213768_s_at'	'230682_x_at'	'ASCL1'	'ABCC3'	3	4
'210727_at'	'230682_x_at'	'CALCA'	'ABCC3'	3	4
'210728_s_at'	'230682_x_at'	'CALCA'	'ABCC3'	3	4
'217495_x_at'	'230682_x_at'	'CALCA'	'ABCC3'	3	4
'202831_at'	'205649_s_at'	'GPX2'	'FGA'	6_2	5_1
'202831_at'	'205650_s_at'	'GPX2'	'FGA'	6_2	5_1
'206343_s_at'	'214774_x_at'	'NRG1'	'TOX3'	5_2	6_1
'206343_s_at'	'215108_x_at'	'NRG1'	'TOX3'	5_2	6_1
'206343_s_at'	'216623_x_at'	'NRG1'	'TOX3'	5_2	6_1
'203438_at'	'205649_s_at'	'STC2'	'FGA'	6_2	5_1
'203438_at'	'205650_s_at'	'STC2'	'FGA'	6_2	5_1
'1554062_at'	'205506_at'	'XG'	'VIL1'	6_2	5_1
'1554062_at'	'228912_at'	'XG'	'VIL1'	6_2	5_1
