# Supplementary material for: Identification of Logic Relationships between Genes and Subtypes of Non-Small Cell Lung Cancer
Source: PLoS One. 2014 Apr 17;9(4):e94644. doi: 10.1371/journal.pone.0094644 (PMC3990524; doi:10.1371/journal.pone.0094644)
Supplement: Table S3 — List of gene-AC/SCC lower and higher logic relationships identified in this paper. (PDF) [file pone.0094644.s006.pdf]

Supporting Information -Table S3: List of gene-AC/SCC lower and higher logic relationships identified in this paper.

Table A 217 gene-AC/SCC lower logic relationships

Series number	Gene	Type (Gene-AC)	Type (Gene-SCC)
1	DST	2	1
2	CLCA2	2	1
3	KRT5	2	1
4	DSG3	2	1
5	GJB5	2	1
6	SERPINB13	2	1
7	S1PR5	2	1
8	BNC1	2	1
9	TRIM29	2	1
10	LOC642587	2	1
11	PKP1	2	1
12	ADAM23	2	1
13	KRT6B	2	1
14	FAT2	2	1
15	GOLT1A	1	2
16	DSC3	2	1
17	NKX2-1	1	2
18	TP63	2	1
19	RORC	1	2
20	LASS3	2	1
21	SMPDL3B	1	2
22	PVRL1	2	1
23	DAPL1	2	1
24	RGL3	1	2
25	NTRK2	2	1
26	COL7A1	2	1
27	FMO5	1	2
28	GBP6	2	1
29	SOX15	2	1
30	SERPINB2	2	1
31	FAM83B	2	1
32	KRT13	2	1
33	ODZ2	2	1
34	ATP11A	1	2
35	KRT16	2	1
36	A2ML1	2	1

37	GJB6	2	1
38	TMEM40	2	1
39	ADH7	2	1
40	TMPRSS11D	2	1
41	HNF1B	1	2
42	GPR39	1	2
43	SPRR3	2	1
44	FAM83C	2	1
45	SLC16A1	2	1
46	ULBP2	2	1
47	SPRR1A	2	1
48	KRT14	2	1
49	COL4A6	2	1
50	PLEKHA6	1	2
51	SPRR2B	2	1
52	VSTM2L	1	2
53	IGSF11	2	1
54	FOXD1	2	1
55	HES2	2	1
56	PAX6	2	1
57	GRHL3	2	1
58	C10orf99	2	1
59	DLX5	2	1
60	STK32A	1	2
61	HOPX	1	2
62	CLDN3	1	2
63	S100A7	2	1
64	GPC1	2	1
65	CAPNS2	2	1
66	C12orf54	2	1
67	WNT2B	2	1
68	NUDT11	2	1
69	C1orf210	1	2
70	BMP7	2	1
71	GJB3	2	1
72	CHRM3	2	1
73	DNALI1	1	2
74	SERPINB5	2	1
75	PI3	2	1
76	LYPD3	2	1
77	LOC146429	1	2
78	MRAP2	2	1
79	PCDH19	2	1

80	LPAR3	2	1
81	FOXL2	2	1
82	DLX1	2	1
83	RNASE7	2	1
84	SH3GL3	2	1
85	STARD5	2	1
86	ABCC6	1	2
87	COL4A3	1	2
88	MMP15	1	2
89	LGSN	1	2
90	FAM181B	2	1
91	SPRR1B	2	1
92	FETUB	2	1
93	ARTN	2	1
94	LMO3	1	2
95	SLC2A1	2	1
96	KRT6A	2	1
97	APOH	1	2
98	PAK7	2	1
99	MSTP9	1	2
100	ARRB1	1	2
101	ARHGEF4	2	1
102	ZFP64	2	1
103	RAPGEFL1	2	1
104	B3GNT6	1	2
105	LRRC31	1	2
106	PITX1	2	1
107	PLEKHB1	1	2
108	EIF5A2	2	1
109	ABCA13	2	1
110	FGFR2	2	1
111	TRIM7	2	1
112	BCL2L15	1	2
113	KRT31	2	1
114	CLCA4	2	1
115	TMPRSS2	1	2
116	ARNTL2	2	1
117	FAM83F	2	1
118	ALPK3	1	2
119	SERPINB4	2	1
120	TNS4	2	1
121	PLD1	2	1
122	ATP1B3	2	1

123	SEMA6D	2	1
124	MICALL1	2	1
125	MARK1	2	1
126	SERPINB3	2	1
127	PHLDB2	2	1
128	LRRN4	1	2
129	LOC145786	1	2
130	BIRC5	2	1
131	SLC6A15	2	1
132	SOX2	2	1
133	PCYT1B	2	1
134	KRTDAP	2	1
135	WFDC5	2	1
136	DLX6	2	1
137	C3orf21	2	1
138	EFCAB4A	1	2
139	GDA	2	1
140	LONRF3	1	2
141	LCE3D	2	1
142	ALOX12	2	1
143	ICA1	1	2
144	KLHDC7A	1	2
145	WDR72	2	1
146	CALML3	2	1
147	GNA14	1	2
148	ARSE	1	2
149	RHCG	2	1
150	VSNL1	2	1
151	WFDC3	1	2
152	SHANK2	1	2
153	LIMCH1	1	2
154	C9orf125	2	1
155	RAB17	1	2
156	BCL11A	2	1
157	DENND2C	2	1
158	KCTD14	1	2
159	ALDH3B1	1	2
160	HOXA1	2	1
161	DMRT3	2	1
162	HOXD13	2	1
163	LOC440335	1	2
164	RMND5A	2	1
165	FOXE1	2	1

166	ACSM3	1	2
167	SBSN	2	1
168	KCNG1	2	1
169	SMO	2	1
170	C3orf34	2	1
171	RBPMS	1	2
172	GALNT10	1	2
173	PRIMA1	2	1
174	B3GNT7	1	2
175	C5orf4	1	2
176	CCNJL	1	2
177	STON2	2	1
178	TMEM125	1	2
179	SPDEF	1	2
180	CXCL2	1	2
181	SFTA2	1	2
182	RNF7	2	1
183	EXO1	2	1
184	RC3H2	2	1
185	GPR87	2	1
186	EN1	2	1
187	ENPP3	1	2
188	SLC44A4	1	2
189	LOC440173	2	1
190	TGM1	2	1
191	SLC1A7	1	2
192	AGR2	1	2
193	TMTC1	2	1
194	C4BPB	1	2
195	CAPN8	1	2
196	DPP4	1	2
197	FST	2	1
198	hCG_1643808	1	2
199	DCUN1D1	2	1
200	IYD	1	2
201	ADA	2	1
202	KCTD15	2	1
203	MST1	1	2
204	CGN	1	2
205	OVGP1	1	2
206	SNCAIP	2	1
207	ATP11B	2	1
208	RHOBTB2	1	2

209	ADCY10	2	1
210	MYBL2	2	1
211	P2RY1	2	1
212	SORBS2	1	2
213	C15orf42	2	1
214	CACNA1D	1	2
215	D4S234E	2	1
216	PLAC2	2	1
217	CABLES1	1	2

Table B 162 gene-AC/SCC higher logic relationships

Series number	Gene1	Gene2	Type (Gene-AC)	Type (Gene-SCC)
1	DUOX2	SORBS2	6_2	5_1
2	PTHLH	FOXA3	6_2	5_1
3	GPX2	FGA	6_2	5_1
4	VSNL1	SNTB1	6_2	5_1
5	CALCA	ABCC3	3	4
6	PLEKHG3	TMEM61	6_2	5_1
7	IRF6	SNTB1	6_2	5_1
8	VSNL1	ZRANB1	6_2	5_1
9	POPDC3	DLK2	4	3
10	TFAP2A	ABCC3	6_2	5_1
11	FOXA3	ABCC3	3	4
12	IRF6	SNTB1	6_2	5_1
13	PTHLH	CALCA	7	8
14	STC2	MESP1	6_1	5_2
15	IRF6	SLC4A4	6_2	5_1
16	FGA	ABCC3	3	4
17	COBL	PGAP1	6_1	5_2
18	GPC1	CFLAR	6_2	5_1
19	B3GNT5	GPC1	2	1
20	IL1F7	PGAP1	6_1	5_2
21	B3GNT5	RGS7	6_2	5_1
22	PTHLH	VIL1	6_2	5_1
23	SORBS2	ARX	3	4
24	FAM62C	LOC100128191	5_1	6_2
25	GPX2	FOXA3	6_2	5_1
26	GAP43	DUOX2	2	1
27	GLB1L3	FOXA3	3	4
28	GPX2	GJB1	6_2	5_1

29	MGC14425	CDC25A	6_1	5_2
30	CFLAR	FLYWCH1	6_1	5_2
31	ITGB4	ABCC3	6_2	5_1
32	PBX1	C11orf41	4	3
33	SEMA3E	DUOX2	6_1	5_2
34	RGS7	ABCC3	3	4
35	PTHLH	DUOX2	2	1
36	ZIC2	IL1F7	6_2	5_1
37	RAPGEF2	PGAP1	6_1	5_2
38	UGT2B4	ABCC3	3	4
39	GJB1	AKR1C2	6_1	5_2
40	GLB1L3	VIL1	3	4
41	DUOX2	CD109	2	1
42	SNRPN	CNTNAP2	6_1	5_2
43	ARHGAP11A	VIL1	6_2	5_1
44	MGC14425	SEMA3E	3	4
45	PTHLH	IL17RB	6_2	5_1
46	WDR66	SLC25A45	5_2	6_1
47	KRT17	UBXN10	6_2	5_1
48	NRG1	LOC730496	5_2	6_1
49	ASCL1	ABCC3	3	4
50	IRF6	BTBD9	6_2	5_1
51	PLEKHG3	WDR1	6_2	5_1
52	PTHLH	PAEP	6_2	5_1
53	CYP2B7P1	DLK2	5_1	6_2
54	ALPK3	PGAP1	6_1	5_2
55	STC2	RHOBTB2	6_2	5_1
56	LOC339988	PGAP1	6_1	5_2
57	TNNC2	RGS7	3	4
58	PLEKHG3	ARX	6_2	5_1
59	VIL1	ABCC3	3	4
60	VSNL1	FGA	6_2	5_1
61	WDR66	CALML5	4	3
62	FAM184A	FOXA3	3	4
63	MUC3B	PCSK9	6_1	5_2
64	PBX1	IL1F9	4	3
65	POF1B	C11orf41	4	3
66	SDK1	SORBS2	6_2	5_1
67	ATP13A3	DUOX2	2	1
68	MUC5B	PIP5KL1	3	4
69	GPX2	SLC2A12	2	1
70	PTHLH	CALCA	7	8
71	GPC1	ABCC3	6_2	5_1

72	TNNC2	SORBS2	3	4
73	UMODL1	ABCC3	3	4
74	CFLAR	ARHGAP23	6_1	5_2
75	GLB1L3	FGA	3	4
76	MUC13	TMEM61	3	4
77	SNRPN	VIL1	3	4
78	TFAP2A	BCL11A	2	1
79	XG	VIL1	6_2	5_1
80	GJB1	DUOX2	6_1	5_2
81	RPTN	MAGEA4	4	3
82	GPX2	MUC13	6_2	5_1
83	TFAP2A	AKR1C2	2	1
84	SEMA3E	PGAP1	6_1	5_2
85	NRG1	TOX3	5_2	6_1
86	STC2	FGA	6_2	5_1
87	COL4A4	DUOX2	6_1	5_2
88	SNRPN	DUOX2	6_1	5_2
89	AP3B2	TFPI2	5_2	6_1
90	COBL	DYNC1I1	6_1	5_2
91	NXPH4	FOXA3	6_2	5_1
92	PTHLH	SNTB1	6_2	5_1
93	SLFN13	C11orf41	5_1	6_2
94	ABCC3	IP6K3	3	4
95	PLEKHG3	CDK6	2	1
96	SPDEF	RGMA	6_1	5_2
97	LOC284578	FGA	3	4
98	CYP4F3	SEMA3E	6_2	5_1
99	AP3B2	ZNF681	5_2	6_1
100	VSNL1	XKRX	6_2	5_1
101	ABCA13	SNTB1	6_2	5_1
102	SNRPN	ARX	3	4
103	ORC1L	DUOX2	2	1
104	TFAP2A	HSPC159	2	1
105	UMODL1	STC2	6_1	5_2
106	ADD2	CALML5	4	3
107	COBL	IPO8	6_1	5_2
108	RHOU	C11orf41	5_1	6_2
109	CFLAR	DUOX2	6_1	5_2
110	GPX2	UBXN10	6_2	5_1
111	ITGB4	HOXD10	2	1
112	C16orf74	LOC145786	5_2	6_1
113	PBX1	POPDC3	4	3
114	CTNND2	ABCC3	3	4

115	DYNC1I1	FGA	6_2	5_1
116	FGA	GAL	6_1	5_2
117	AP3B2	LRP2	5_2	6_1
118	DOK1	C11orf41	5_1	6_2
119	TNNC2	C11orf41	6_1	5_2
120	WDR66	RAF1	5_2	6_1
121	GPC1	CDK6	2	1
122	TNNC2	FGA	3	4
123	IRF6	COBL	6_2	5_1
124	RHOBTB2	SETD8	6_1	5_2
125	DQX1	ZNF681	5_2	6_1
126	PTHLH	CITED4	2	1
127	FOXA2	DUOX2	6_1	5_2
128	IRF6	GSDMC	2	1
129	GPX2	ITGB8	2	1
130	CYP2B7P1	MAGEA4	5_1	6_2
131	CITED4	PGAP1	2	1
132	ZNF556	TDRKH	5_2	6_1
133	HTR2C	C11orf41	4	3
134	LOC283485	HABP2	5_2	6_1
135	MRPS12	RBPMS2	6_2	5_1
136	FGA	AKR1C2	6_1	5_2
137	C1orf110	C11orf41	4	3
138	ARX	CD109	6_1	5_2
139	RGS7	CFLAR	3	4
140	ARHGAP11A	VIL1	6_2	5_1
141	ATP13A3	VIL1	6_2	5_1
142	HPN	MAGEA4	5_1	6_2
143	SYT14	LOC730496	5_2	6_1
144	ZNF639	MGC10981	5_2	6_1
145	TFAP2A	IL1F7	6_2	5_1
146	PTHLH	PSG6	6_2	5_1
147	ALPK3	SLC25A45	3	4
148	VSNL1	FGF9	6_2	5_1
149	B3GNT5	GSDMC	2	1
150	SPDEF	H2AFY2	6_1	5_2
151	ITGA3	MGC10981	1	2
152	PTHLH	ZRANB1	6_2	5_1
153	COBL	B3GNT5	6_1	5_2
154	VSNL1	CFLAR	6_2	5_1
155	PBX1	CNFN	4	3
156	AQP4	GOLGA2L1	1	2
157	C9orf103	C11orf41	5_1	6_2

158	NRG1	C16orf89	5_2	6_1
159	VIL1	COL4A3	3	4
160	ATP8A1	IL1F7	3	4
161	H2AFY2	PGAP1	2	1
162	CFLAR	VIL1	3	4
