# Supplementary material for: Identification of Logic Relationships between Genes and Subtypes of Non-Small Cell Lung Cancer
Source: PLoS One. 2014 Apr 17;9(4):e94644. doi: 10.1371/journal.pone.0094644 (PMC3990524; doi:10.1371/journal.pone.0094644)
Supplement: Table S6 — Gene pairs related with AC or SCC through the logic function AND or XOR. (PDF) [file pone.0094644.s009.pdf]

Supporting Information -Table S6: Gene pairs related with AC or SCC through the logic function AND or XOR

Gene1	Gene2	Type (Gene-AC)	Type (Gene-SCC)
ITGA3	MGC10981	1	2
AQP4	GOLGA2L1	1	2
B3GNT5	GPC1	2	1
GAP43	DUOX2	2	1
PTHLH	DUOX2	2	1
DUOX2	CD109	2	1
ATP13A3	DUOX2	2	1
GPX2	SLC2A12	2	1
TFAP2A	BCL11A	2	1
TFAP2A	AKR1C2	2	1
PLEKHG3	CDK6	2	1
ORC1L	DUOX2	2	1
TFAP2A	HSPC159	2	1
ITGB4	HOXD10	2	1
GPC1	CDK6	2	1
PTHLH	CITED4	2	1
IRF6	GSDMC	2	1
GPX2	ITGB8	2	1
CITED4	PGAP1	2	1
B3GNT5	GSDMC	2	1
H2AFY2	PGAP1	2	1
PTHLH	CALCA	7	8
